# Features from the photoplethysmogram and the electrocardiogram for estimating changes in blood pressure

**DOI:** 10.1038/s41598-022-27170-2

**Published:** 2023-01-18

**Authors:** Eoin Finnegan, Shaun Davidson, Mirae Harford, Peter Watkinson, Lionel Tarassenko, Mauricio Villarroel

**Affiliations:** 1 Institute of Biomedical Engineering, Department of Engineering Science, University of Oxford, Oxford, UK; 2 Critical Care Research Group, Nuffield Department of Clinical Neurosciences, University of Oxford, Oxford, UK; 3 NIHR Oxford Biomedical Research Centre, Oxford, UK

**Keywords:** Cardiovascular biology, Predictive markers, Cardiovascular diseases

## Abstract

There is a growing emphasis being placed on the potential for cuffless blood pressure (BP) estimation through modelling of morphological features from the photoplethysmogram (PPG) and electrocardiogram (ECG). However, the appropriate features and models to use remain unclear. We investigated the best features available from the PPG and ECG for BP estimation using both linear and non-linear machine learning models. We conducted a clinical study in which changes in BP ($\Delta$BP) were induced by an infusion of phenylephrine in 30 healthy volunteers (53.8% female, 28.0 (9.0) years old). We extracted a large and diverse set of features from both the PPG and the ECG and assessed their individual importance for estimating $\Delta$BP through Shapley additive explanation values and a ranking coefficient. We trained, tuned, and evaluated linear (ordinary least squares, OLS) and non-linear (random forest, RF) machine learning models to estimate $\Delta$BP in a nested leave-one-subject-out cross-validation framework. We reported the results as correlation coefficient ($\rho _p$), root mean squared error (RMSE), and mean absolute error (MAE). The non-linear RF model significantly ($p<0.05$) outperformed the linear OLS model using both the PPG and the ECG signals across all performance metrics. Estimating $\Delta$SBP using the PPG alone ($\rho _p$ = 0.86 (0.23), RMSE = 5.66 (4.76) mmHg, MAE = 4.86 (4.29) mmHg) performed significantly better than using the ECG alone ($\rho _p$ = 0.69 (0.45), RMSE = 6.79 (4.76) mmHg, MAE = 5.28 (4.57) mmHg), all $p < 0.001$. The highest ranking features from the PPG largely modelled increasing reflected wave interference driven by changes in arterial stiffness. This finding was supported by changes observed in the PPG waveform in response to the phenylephrine infusion. However, a large number of features were required for accurate BP estimation, highlighting the high complexity of the problem. We conclude that the PPG alone may be further explored as a potential single source, cuffless, blood pressure estimator. The use of the ECG alone is not justified. Non-linear models may perform better as they are able to incorporate interactions between feature values and demographics. However, demographics may not adequately account for the unique and individualised relationship between the extracted features and BP.

## Introduction

Changes in the cardiovascular and autonomic nervous systems are reflected in changes in signals such as the photoplethysmogram (PPG) and the electrocardiogram (ECG). These physiological signals are ubiquitous in a clinical setting and increasingly in an out-of-clinic setting due to the development of wearables such as smartwatches. As a result, recent advances in non-invasive, cuffless estimation of blood pressure (BP) have been focused on utilising the PPG and the ECG signals. For example, pulse arrival time (PAT), computed as the time difference between two fiducial points in the ECG and PPG waveforms, has been shown in certain studies to have strong correlations with BP^1^. However, measuring PAT requires two synchronous devices, both of which are susceptible to independent sources of noise such as motion artefacts. Additionally, factors such as the pre-ejection period (PEP) (the time delay between the electrical depolarisation of the heart’s left ventricle and the opening of the aortic valve) may impact PAT estimates independently of BP^2^. A summary of BP estimation methods using PAT can be found in^3–5^.

Driven by modern techniques, research on cuffless BP estimation has increasingly focused on relating morphological features of the PPG and the ECG waveforms to BP (or changes in BP, $\Delta$BP) using data-driven models. However, the optimal features and models required for accurate BP estimation remain unclear. In this paper, we implemented linear and non-linear machine learning (ML) models to estimate $\Delta$BP using a large and diverse cohort of features from the PPG and the ECG waveforms. In addition to features commonly used in the literature, we proposed new features from both signals and assessed their individual importance for estimating $\Delta$BP using Shapley additive explanation values. We compared our results to those estimating changes in BP using PAT, and evaluated the PPG and ECG as potential single source devices for BP estimation. This work was carried out using data from a clinical study involving 30 healthy volunteers. Changes in BP were induced by the administration of phenylephrine, a vasoactive medication that causes arterial and venous vasoconstriction and increases cardiac preload (initial stretching of cardiac muscles).

### Relationship between changes in BP and changes in the PPG

The pulsatile PPG waveform is related to changes in blood volume over time in a bed of tissue^6^. The PPG signal is typically recorded by a pulse oximeter placed on the index finger. Methods using the PPG have been proposed as a continuous, non-invasive, cuffless approach to estimate BP^7^. The PPG–BP relationship is driven, in part, by the theoretical relationship between changes in pressure and volume of blood in a localised region of the arteries^7^, as well as the impact of reflected pressure waves^8^. Reflected pressure waves result at points of significant impedance mismatch further down the arterial tree and travel back to the point of PPG measurement. The first reflection site is at the junction of the renal arteries resulting in a reflected pressure wave (known as the tidal wave) that is typically present in late systole^9^. The second reflection site is at the junction of the iliac arteries resulting in a reflected pressure wave (known as the dicrotic wave) that is typically present in early diastole^10^. Changes in arterial stiffness are thought to have a significant impact on the timing, amplitude, and morphology of the reflected pressure waves and therefore may significantly impact the shape of the PPG waveform^8^. The PPG offers significant benefits for BP monitoring over conventional cuff-based measurements. Most notably, the PPG can be recorded by a single, unobtrusive optical sensor which also has the potential to be implemented on a wearable device such as a smartwatch^11,12^. However, there is no generally accepted method relating changes in the PPG waveform to $\Delta$BP and, as a result, a variety of different approaches are proposed in the literature. A summary of BP estimation algorithms using the PPG waveform can be found in^7,13,14^.

Much like PAT^1^, features extracted from the PPG waveform are thought to be subject-specific requiring calibration for accurate mapping to BP values^7^. Mukkamala et al.^7^ splits calibration strategies into three groups: *individual*, *hybrid* and *population*. In *individual calibration*, all model parameters are determined by multiple paired recordings of BP and PPG from a single individual. This approach may be feasible for BP estimation using PAT (where typically only two parameters are required for modelling^1^), however it becomes intractable for multi-parameter ML models often employed for PPG-based BP estimation. In *hybrid calibration*, only one calibration BP data point is required for a single individual. The remaining model parameters are estimated using the individual’s demographics and a training set comprised of multiple BP-PPG pairs from a cohort of different individuals. In *population calibration*, no calibration recordings are required and all calibration is handled using the individual’s demographics and a similar training set. Certain dependencies on the morphology of the PPG waveform have been previously reported for age^15^, sex^16^ and body mass index (BMI)^17^, however these are often not strong enough to allow for good accuracy when using *population calibration* strategies. As a result, the majority of studies proposing PPG-based BP estimation opt for a *hybrid calibration* strategy.

Sun et al.^18^ evaluated the use of a linear regression model for estimating systolic blood pressure (SBP) measured by a commercial Portapres device (Finapres), using the volume-clamp method. Nineteen subjects underwent an exercise test followed by a posture change test. Nineteen features were extracted from the PPG. Using a *hybrid calibration* strategy, the authors reported a root mean squared error (RMSE) of 8.99 mmHg and Pearson’s correlation coefficient ($\rho_{p}$) of 0.85 during the exercise test, and a RMSE of 7.33 mmHg and $\rho_{p}$ of 0.47 during the posture change test. Normalised weights of the linear model were used to highlight features that had the most predictive power and an inconsistency of the best features was found between the two tests. Miao et al.^19^ used support vector regression on 14 extracted features from 73 subjects to track BP changes induced by physical exercise. A genetic algorithm for feature selection was developed to highlight features that best estimated $\Delta$BP. The stability of the proposed models was evaluated in a follow-up study for 1 day, 10 days and 6 months after the initial test. The results suggested that, similar to PAT^20,21^, the models lose their accuracy over time and therefore require frequent recalibration. Hasanzadeh et al.^22^ extracted features from the PPG in a subset of 1,000 individuals from the MIMIC-III dataset. The intra-arterial blood pressure was used as a reference. The authors implemented a linear regression model as well as a non-linear tree-based model, AdaBoost. AdaBoost significantly outperformed the linear model for their dataset. Additionally, the authors highlighted the sensitivity of PPG feature detection in the presence of random noise. Recent shifts in the field have moved towards the application of deep learning algorithms^23,24^ which often do not require the extraction of handcrafted features and instead work on the raw PPG waveform. However, while these approaches are reported to improve the estimation accuracy, they lose out in model interpretability due to their black-box nature.

### Relationship between changes in BP and changes in the ECG

The ECG is a measure of the electrical activity generated in the myocardium (heart muscle) during each heartbeat. It is acquired by measuring the voltage difference between two points on the body surface over time^25^. A single lead ECG is typically recorded by three electrodes placed on an individual’s torso forming Einthoven’s triangle^25^.

In comparison to the PPG, less focus has been placed on the potential use of the ECG for BP estimation. The general theory governing the relationship between ECG and BP is based on a cyclical process known as mechano-electric coupling (MEC)^26^. Changes in the electrical properties of the heart have a direct impact on its contractility. This is known as excitation-contraction coupling. Similarly, changes in the mechanical properties of the tissues surrounding the heart are detected by mechanosensitive ion channels, resulting in local changes in the electrical potential. This is known as mechano-electrical feedback. MEC, therefore, describes the cyclical process whereby changes in the ECG waveform can reflect $\Delta$BP. However, MEC is influenced not only by extra-cardiac control mechanisms such as the Autonomic Nervous System (ANS) and hormonal changes, but also by environmental mechanisms such as ion concentrations and temperature^27^. Therefore, the relationship between $\Delta$BP that can be detected by analysing morphological changes in the ECG waveform are not as developed as methods using the PPG waveform.

Simjanoska et al.^28^ used a dataset containing 51 individuals from a mixture of 4 open-source datasets. Three of the datasets (totalling 44 individuals) included healthy volunteers using commercial ECG sensors with reference BP values measured by a cuff. In these datasets BP was perturbed by natural variations with each individual contributing a range from 1 to 8 measurements overall. The fourth dataset was recorded from 7 patients with traumatic brain injuries in an intensive care unit (ICU) using clinical-grade ECG sensors and reference BP values measured by an arterial line. Complexity features such as mobility and entropy were extracted from 30-second segments of ECG data and used to train a random forest regression model using a train-validation-test split. After calibration, the model achieved mean absolute error (MAE) of 7.72 mmHg for SBP, 9.45 mmHg for diastolic blood pressure (DBP), and 8.13 mmHg for mean arterial blood pressure (MAP). However, the authors only implemented a small number of complexity features, and the mixture of data sources (especially with regard to mixing healthy and unhealthy populations) makes interpreting the results difficult.

## Methods

### Clinical study

Thirty healthy volunteers with no history of cardiovascular disease were recruited for the clinical study. The study protocol has been outlined previously^1,29^. The study took place at the Cardiovascular Clinical Research Facility within the John Radcliffe Hospital, Oxford, UK. This study was reviewed and approved by the Oxford University Research and Ethics Committee and Clinical Trials and Research Governance teams (R63796/RE001). All methods were performed in accordance with the relevant guidelines and regulations. Individual informed written consent was obtained from all the participants in the study to record the data and publish the results.

The study was split into four main stages: rest, dose increase, maximum infusion, and washout. Each session began with a 5-minute resting period. Participants were then administered an infusion of phenylephrine. Phenylephrine is an $\alpha _1$ adrenergic receptor agonist which causes arterial and venous vasoconstriction as well as reflex bradycardia (slowing down of heart rate)^30^. The dosing regimen was guided by the clinician in the Medical Sciences Division Ethics Committee (University of Oxford) who balanced the desired clinical effect against any safety concerns^29^. Phenylephrine was administered as an intravenous solution, starting at a rate of 0.2mcg/kg/min with an increase of 0.2mcg/kg/min every 1 minute for 10 increments. Once the maximum rate was reached, the infusion and all monitoring remained constant for a further 6 minutes. Each session ended with an 8-minute washout period. Participants were asked to refrain from ingesting caffeinated drinks for four hours prior to the study visit as caffeine is a vasoconstrictor. Participants lay with the head and trunk raised to between 15 and 45 degrees (semi-Fowler’s position).

#### Instrumentation

A Philips Intellivue MX800 patient monitor (Philips, Netherlands) recorded BP via a sphygmomanometer cuff. The Philips comfort care M3001A BP cuff was wrapped around the upper left arm of the participant with the centre directly above the brachial artery as recommended^31^. The ixTrend software (Ixellence GmbH, Germany) was used to record the data generated by the patient monitor. A Stowood Visi Black shadow polygraphy device (Stowood, UK) recorded the ECG and PPG signals. Three ECG electrodes were attached to the participant’s torso forming Einthoven’s triangle. A pulse oximeter (Masimo, USA) probe was placed on the participant’s right hand. Table 1 provides a summary of the physiological parameters recorded by the Philips and Stowood monitors.

**Table 1:** Physiological parameters and sampling rates recorded by the two devices used in this study.

Device	Name	Description	Sampling rate (Hz)
Philips	SBP, MAP, DBP	Blood pressure signal from the sphygmomanometer cuff	1/60
Stowood	ECG PPG HR	1-lead electrocardiography signal Photoplethysmography signal Heart rate derived from Masimo pulse oximeter	256 512 5

### Features from the PPG

To preprocess the PPG waveform, we performed the following steps. The PPG signal was first filtered using an 8^th^-order Butterworth infinite impulse response (IIR) band-pass filter with cut-off frequencies of 0.5 Hz and 10 Hz as recommended in^3^. We then followed the work of Villarroel et al.^32^ to detect the pulse onsets and to assess the quality of the PPG signal (SQI_PPG_). Amplitude modulation of the PPG can result from respiration^33^ as well as changes in contact pressure of the sensor on the surface of the skin^34^. In order to reduce the dependence of this amplitude modulation as well as heart rate, we normalised the PPG beats to have unit amplitude and time duration. The first and second derivatives of the PPG beats, known as the velocity plethysmogram (VPG) and acceleration plethysmogram (APG) respectively, were extracted using a 7^th^-order Savitzky-Golay filter^35^. We used the nomenclature defined by Elgendi et al.^36^ for the characteristic fiducial points of the PPG, VPG, and APG.

**Figure 1:**
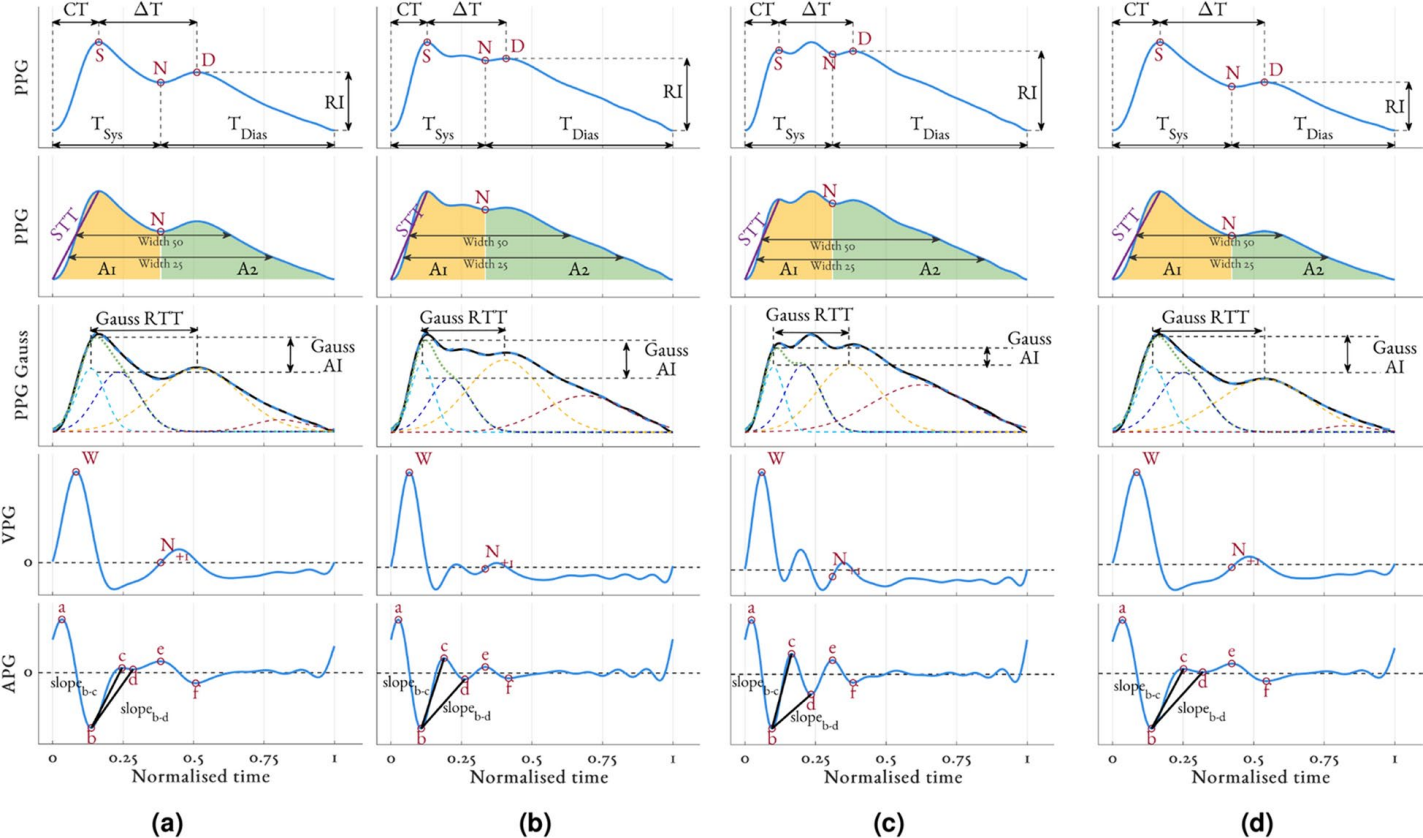
Overview of the fiducial points detected and some of the features extracted from the PPG waveform for one individual (Male, Age: 24, BMI: 25.1) during the four stages of the study protocol: (**a**) rest, (**b**) dose increase, (**c**) maximum infusion, and (**d**) washout. Examples of the following features are provided: Crest time (CT), $\Delta$T, reflection index (RI), width_25_, width_50_, slope transit time (STT), A1, A2, Gaussian estimation of the transit time of the reflected wave (Gauss RTT) and augmentation index (Gauss AI), slope_b-c_ and slope_b-d_. Acronyms: S - Systolic peak, N - Dicrotic notch, D - Diastolic peak, a-e - waves of the APG.

Figure 1 shows the typical changes in the morphology of the PPG, VPG and APG beat seen for a participant during the four main stages of the study protocol (rest, dose increase, maximum infusion and washout). The following fiducial points were detected using the criteria defined in^37^: dicrotic notch (N), diastolic peak (D) and the *a*, *b*, *c*, *d*, and *e* waves on the APG. The dicrotic notch is produced by the closing of the aortic valve and therefore marks the end of systole and the beginning of diastole. The systolic peak (S) was defined as the first turning point of the PPG pulse above its midpoint.

We identified features from previous publications describing methods for estimating $\Delta$BP from the PPG. Table 2 provides a summary of the features we extracted from the PPG. We grouped these features into five main categories: PPG morphology, VPG morphology, APG morphology, Gaussian decomposition, and principal component analysis (PCA).

### PPG morphology

As BP is determined by the product of total peripheral reistance (TPR) and cardiac output (CO), features reflecting variations in these two metrics are likely to provide a strong indication of $\Delta$BP.

Changes in TPR can significantly impact the morphology of the PPG through interactions with the incident forward travelling wave produced by left ventricular contraction and the reflected pressure waves^8^. To model the varying amplitude of the reflected pressure waves, we included the dicrotic notch amplitude ($\hbox {N}_{amp}$), and the diastolic peak amplitude (labelled reflection index, RI, for consistent feature notation with the literature) as features^38^. As the tidal and dicrotic reflected waves typically arrive during systole and diastole respectively, Lyu et al.^39^ proposed the stress-induced vascular response index (sVRI) as the ratio of the mean of the PPG in the diastolic phase, $\mu$_Dias_, to the mean of the PPG in the systolic phase, $\mu$_Sys_. This effect was additionally modelled using the areas under PPG during the systolic (A1) and diastolic (A2) phases^40^. The ratio of these two areas (A2/A1) is referred to as the inflection point area ratio (IPA).

The influence of the reflected pressure waves was additionally modelled through analysis of the frequency domain by assessing the level of distortion in the PPG waveform as the relative power of its harmonic frequencies using the normalised harmonic area (NHA)^40^.

The increased velocity of the reflected waves was modelled by relative time delays such as $\Delta$T (time between *S* and *D*)^41^, crest time (CT, time from onset to *S*)^15^, T_Sys_ (time in systolic phase), and T_Dia_ (time in diastolic phase), and T_Ratio_ (T_Sys_/T_Dia_)^42^. Additionally, the time delay between the systolic peak and dicrotic notch is related to the transit time of the dicrotic wave to the site of reflection and back. Shin et al.^43^ suggest that this time delay may be calibrated to the individual by normalising by the time delay between the maximum derivative of the PPG and the dicrotic notch, and multiplying the resultant by the individual’s height. This feature is labelled pressure index (PI). Lin et al.^44^ recently suggested that these time delay features may additionally reflect changes in CO. CO monitoring using the PPG has been demonstrated using the inflection and harmonic area ratio (IHAR)^40^.

A small number of features parameterise changes in the overall PPG morphology. For example, $\hbox {Width}_{25}$ and $\hbox {Width}_{50}$ track variations in the width of the PPG at 25% and 50% of the overall amplitude respectively^41,45^. Skewness and kurtosis both characterise the distribution of the PPG beat values^23^. Kurtosis is a measure of the relative “tailness” of the distribution. Skewness is a measure of the asymmetry of the data around the mean.

### VPG morphology

We included four features derived from analysing the VPG morphology. In order to model the changing velocity of the pulse wave during the two main phases of the cardiac cycle, we included the mean and standard deviation of the VPG during systole ($\hbox {Sys}_\mu$ and $\hbox {Sys}_\sigma$ respectively) and diastole ($\hbox {Dia}_{\mu }$ and $\hbox {Dia}_\sigma$ respectively)^18^.

### APG morphology

We included eight features derived from analysing the APG morphology. The amplitude of each APG wave, normalised by the amplitude of *a*, exhibits a moderate linear relationship to age^46^. *b*/*a* increase with age, whereas *c*/*a*, *d*/*a*, and *e*/*a* decrease with age. These relationships were implemented into a single feature using the ageing index (AGI) $(b-c-d-e)/a$^46^. In addition, Mok Ahn et al.^42^ proposed the gradient of the straight lines joining the *b* wave to the *c* and *d* wave (slope_b-c_ and slope_b-d_ respectively) as additional indicators of age-related arterial stiffness. Finally, Pilt et al.^47^ suggested that the PPG waveform index (PPG AI), computed as the ratio of PPG waveform coincident with the *d* wave of the PPG (labelled $d_{-2}$) to the ratio of PPG waveform coincident with the *b* wave of the PPG (labelled $b_{-2}$), reliably tracks changes in arterial stiffness.

**Table 2:** Summary of features extracted from the PPG.

Category	Feature notation and publication	Description or formula
PPG morphology	Notch amplitude ($\hbox {N}_{amp}$)	Amplitude of the dicrotic notch (see Fig. 1)
	Reflective index (RI)^38^	Amplitude of the diastolic peak (see Fig. 1)
	$\Delta$ T^41^	Time from systolic peak to diastolic peak (see Fig. 1)
	Crest Time (CT)^15^	Time from onset to systolic peak (see Fig. 1)
	T_Sys_^42^	Time in systolic phase (see Fig. 1)
	T_Dia_^42^	Time in diastolic phase (see Fig. 1)
	T_Ratio_^42^	T_Sys_/T_Dia_
	Slope transit time (STT)^50^	Slope of straight line from onset to peak (see Fig. 1)
	Stress-Induced Vascular Response Index (sVRI)^39^	$\mu$_Dias_/$\mu$_Sys_
	A1^40^	Area under PPG in systolic phase (see Fig. 1)
	A2^40^	Area under PPG in diastolic phase (see Fig. 1)
	Inflection point area (IPA)^40^	A2/A1
	$\hbox {Width}_{25}$^41^	Width of the PPG at 25 % of its amplitude (see Fig. 1)
	$\hbox {Width}_{50}$^41^	Width of the PPG at 50 % of its amplitude (see Fig. 1)
	Pressure index (PI)^43^	$\frac{t(N) - t(S)}{t(N) - t(W)} \times h$
	Normalised harmonic area (NHA)^40^	$\sum _{n=2}^{N}FFT^2(f_n)/\sum _{n=1}^{N}FFT^2(f_n)$
	Inflection and harmonic area ratio (IHAR)^40^	(1-NHA)/ IPA
	Skewness^23^	–
	Kurtosis^23^	–
VPG morphology	$Sys_\mu$^18^	Mean of VPG in the systolic phase
	$\hbox {Sys}_\sigma$^18^	Variance of VPG in the systolic phase
	$\hbox {Dia}_{\mu }$^18^	Mean of VPG in the diastolic phase
	$\hbox {Dia}_\sigma$^18^	Variance of VPG in the diastolic phase
APG morphology	$\frac{b}{a}$, $\frac{c}{a}$, $\frac{d}{a}$ & $\frac{e}{a}$^46^	
	Ageing index (AGI)^46^	$\frac{b - c - d - e}{a}$
	slope_b-c_^42^	Slope of a straight line between *b* and *c*, normalised by *a* (see Fig. 1)
	slope_b-d_^42^	Slope of a straight line between *b* and *d*, normalised by *a* (see Fig. 1)
	PPG AI^47^	PPG augmentation index $d_{-2}/b_{-2}$
Gaussian decomposition	$\hbox {A}_{g1-4}$, $\sigma _{g1-4}$, $\mu _{g1-4}$	Amplitude, variance and mean of the four decomposed Gaussians
	Gaussian augmentation index (Gauss AI)^12^	$\max (g_s) - A_{g3}$ (see Fig. 1)
	Gaussian reflection index (Gauss RI)^12^	$\sum (g_s) - \sum (g_3)$
	Gaussian reflected wave transit time (Gauss RTT)^48^	$\mu _{g3} - \mu _{g1}$ (see Fig. 1)
	Gaussian augmentation index_R_ (Gauss AI_R_)^48^ $$^{\dagger }$$	$$\frac{A_{g1} - A_{g2}}{A_{g1}}$$
	Gaussian reflection index_R_ (Gauss RI_R_)^48^ $$^{\dagger }$$	$$A_{g3}/A_{g1}$$
	Gaussian approximation of left ventricular ejection time (Gauss LVET)^51^	See reference for definition
	$\hbox {Gauss}_{Sys/Dias}$ *	$\sum (g_s)/\sum (g_d)$
	$\hbox {Gauss}_{A4/A1}$ *	$A_{g4}/A_{g1}$
	$\hbox {Gauss}_{\sigma 4/A1}$ *	$\sigma _{g4}/A_{g1}$
PCA	PPG PCA _1-3_^52^	First 3 principal components of PPG beat
	VPG PCA _1-3_^52^	First 3 principal components of VPG beat
	APG PCA _1-3_^52^	First 3 principal components of APG beat

*Indicates features that, to the authors’ knowledge, have not been previously implemented for BP estimation. $^{\dagger }$ Note that there are two Gaussian indices referenced reflection index and augmentation index. We refer to the second set using the subscript R to reflect the authors: Rubins et al. $\mu _{\textrm{Sys}}$ and $\mu _{\textrm{Dias}}$ represents the mean of the PPG during systole and diastole respectively. *h* is the height of the participant. *t* is the normalised time since the pulse onset. FFT is the fast Fourier transform. $g_i$ refers to the $i^{\textrm{th}}$ Gaussian; $g_s$ = $g_1$ + $g_2$ representing the systolic wave; $g_d$ = $g_3$ + $g_4$ representing the diastolic wave. $\sum (g_i)$ is the area under the $i^{\textrm{th}}$ Gaussian.

#### Gaussian decomposition

Each PPG pulse (of unit amplitude and duration) was decomposed into the summation of four Gaussians^48^. This approach has the advantage of providing a representation of the PPG pulse without reliance on fiducial point detection. Additionally, it allows for the modelling of reflected wave interactions which are thought to have a Gaussian profile^49^.

For a PPG pulse $\zeta$ we computed the modelled pulse, $\zeta$^Gauss^, as:

1 $$\begin{aligned} \zeta ^{Gauss}(t, \Theta ) = \sum _{i=1}^4 g_i(t, \theta _i) \end{aligned}$$

where $g_i$ represents the *i*^th^ Gaussian component modelled as:

2 $$\begin{aligned} g_i(t, \theta _i) = A_{gi} \times e^{-\frac{(t-\mu _{gi})^2}{2\sigma _{gi}^2}} \end{aligned}$$

where *t* is the normalised time duration for the PPG pulse, and $\theta _i$ is a vector, [$A_{gi}$, $\mu _{gi}$, $\sigma _{gi}$], containing the respective amplitude, mean and variance of each Gaussian. $\Theta$ = [$\theta _1$, $\theta _2$, $\theta _3$, $\theta _4$] and thus Gaussian decomposition parameterises each PPG beat into 12 components. To determine the optimum value for $\Theta$, $\hat{\Theta }$, we implemented a bounded Levenberg-Marquart optimisation algorithm to minimise the root mean squared error loss, *L*^Gauss^, between $\zeta$ and $\zeta$^Gauss^ given as:

3 $$\begin{aligned} L^{\text {Gauss}} (\Theta ) = \sqrt{\frac{1}{N}\sum (\zeta -\zeta ^{\text {Gauss}}(t, \Theta ))^2} \quad , \qquad \hat{\Theta } = \mathop {\textrm{argmin}}\limits _{\Theta } ( L^{\text {Gauss}} (\Theta )) \end{aligned}$$

The optimisation was bounded such that all parameters were positive and the amplitudes were all less than 1. Additionally, $\{\mu _1, \mu _2,\mu _3,\mu _4\}$ must be a monotonically increasing sequence. *L*^Gauss^ is non-convex and therefore the optimised values were dependent on initial conditions. For the first beat, the initial conditions were: $\theta _1 = [0.9, 0.2, 0.01]$, $\theta _2 = [2/3 , 0.4, 0.01]$, $\theta _3 = [0.5, 0.6, 0.01]$, and $\theta _4 = [1/3, 0.8, 0.01]$. These parameters resulted in an evenly distributed set of Gaussians with equal widths. The amplitudes were determined in order to match the contour of a typical PPG beat. To encourage continuity of parameters from beat-to-beat, we used the optimised parameters for the previous beat as initial seeds for the optimisation of the current beat. Following the work of^53^, we set the SQI_PPG_ of each beat to 0 if the value of $L^{\text {Gauss}} (\hat{\Theta })$ for that beat was greater than 0.03.

From pulse decomposition analysis^8^, $g_1$ represents the incident forward travelling pressure wave produced by left ventricular contraction. $g_2$ represents the tidal wave^9^. $g_2$ is typically observed during systole, therefore $g_s = g_1 + g_2$ reflects the systolic component. $g_3$ represents the dicrotic wave^10^. $g_4$ represents the additional minor reflections and re-reflections in the systemic vascular structure with lower amplitude than the two main reflected waves^54^. $g_3$ and $g_4$ are both typically dominant in the diastole phase, therefore $g_d = g_3 + g_4$ reflects the diastolic component.

Table 2 provides a summary of the Gaussian decomposition features used. Together with the values of $\hat{\Theta }$, we implemented various features derived from the Gaussian decomposition that have been previously proposed as indicators of arterial stiffness in the literature^48,51^. Additionally, through observations of Gaussian decomposition in our dataset, we propose three new features: the ratio of the systolic component to the diastolic component ($\hbox {Gauss}_{Sys/Dias}$); the amplitude of the fourth Gaussian scaled by the amplitude of the first Gaussian ($\hbox {Gauss}_{A4/A1}$); and the variance of the fourth Gaussian scaled by the amplitude of the first Gaussian ($\hbox {Gauss}_{\sigma 4/A1}$) (scaling by variance rather than the amplitude of the first Gaussian gave a less informative parameter). Figure 1 shows an example of Gaussian decomposition for a typical participant during the four main stages of the study protocol: rest, dose increase, maximum infusion, and washout. Figure 1 also presents examples of feature extraction for Gaussian estimation of the transit time of the reflected wave (Gauss RTT) and augmentation index (Gauss AI).

#### Principal components

Principal component analysis (PCA)^55^ maps high-dimensional data to a lower dimension along orthogonal principal components. These principal components account for the majority of the variation in the original data and therefore highlight regions of significant change in the PPG, VPG and APG signals. We computed PCA features using the following steps:

1. Resample all good-quality beats (defined as an SQI_PPG_ $> 0.8$) from the PPG, VPG and APG signals to be 100 samples in length using cubic spline interpolation.
2. Pool all resampled PPG, VPG and APG beats from all participants to form 3 matrices: $\Psi$_PPG_, $\Psi$_VPG_ and $\Psi$_APG_ respectively.
3. Mean normalise each $\Psi$ matrix.
4. Perform PCA on each $\Psi$ independently by computing the eigenvectors of the corresponding covariance matrix and extract the first 3 principal components that correspond to the largest eigenvalues.

We extracted 3 principal components as this was found empirically to explain more than 85% of the variation in the $\Psi$_PPG_, $\Psi$_VPG_ and $\Psi$_APG_ datasets. A visualisation of the computed PCA eigenvectors is provided in Supplementary Information figure SI: 1.

### Features from the ECG

To preprocess the ECG waveform, we performed the following steps. To suppress the impact of baseline wander, the ECG was filtered using an 8$^{\text {th}}$-order Butterworth IIR high-pass filter with a cut-off frequency of 0.5 Hz. To suppress power-line interference, a 2^nd^-order IIR notch filter with centre frequency at 50 Hz (the frequency of mains power in the UK) was used. We detected the QRS complex following the work of Pan and Tompkins^56^ and assessed the quality of the ECG (SQI_ECG_) following the work of Li et al.^57^.

The features we extracted from the ECG are summarised in table 3. Features relating to complexity and entropy of the ECG have been previously proposed to track changes in BP^28,58^. These features quantify the level of regularity and unpredictability of fluctuations over a time series. Generally, a higher-level complexity indicates a more irregular dynamic system. A lower-level complexity indicates the presence of central trends or cyclical patterns. Changes in entropy of the ECG time series have been shown to track changes in heart rate variability (HRV) caused by myocardial ischaemia^59^ and also denote periods of cardiac arrhythmia^60^. We provide full details of the algorithms used in Supplementary Information SI: 2.

**Table 3:** Summary of features extracted from the ECG.

Feature notation and publication	Description or formula
Hjorth mobility^28^	Estimate of the signal’s mean frequency (Equation SI 1)
Hjorth complexity^28^	Estimate of the signal’s bandwidth (Equation SI 2)
Fractal dimension^28^	Computed using Higuchi’s algorithm^61^ with $k_{max} = 17$
Shannon entropy (SE)^28^	Uncertainty of information content based on probability distribution (Equation SI 3)
Approximate entropy (approxEnt) *	Quantifies regularities of signal
Sample entropy (sampEnt) *	approxEnt computed without self comparisons
Multi-level sample entropy (MSE) *	Course approximations of sample entropy, computed at scales 2, 4, 6, 8

* Indicates features that, to the authors’ knowledge, have not been previously implemented for BP estimation. Full details of the algorithms and equations used for computing each feature can be found in Supplementary Information SI: 2.

### Pulse arrival time

PAT has been shown previously to provide a beat-by-beat estimate of changes in arterial stiffness and therefore may be a good surrogate for BP^1^. PAT and its corresponding SQI_PAT_ was computed in the same manner as we have previously reported^1^. We used PAT in a baseline model to compare the performance of PPG and ECG features for BP estimation.

### Computing the reference BP values

Measurements of SBP, MAP, and DBP using sphygmomanometer cuffs have known limitations depending on posture, cuff-inflation hypertension and cuff size^62,63^. The sphygmomanometer cuff used in our study was programmed to inflate every minute. However, errors in cuff inflation prevented the Philips monitor from registering an accurate estimate, resulting in a missed data-point in the recorded BP time series. Therefore, data from the cuff was both noisy and sparse. In order to reduce the impact of these sources of error, we processed the cuff data using a cubic smoothing splines^64^ algorithm. This allowed for both filtering and interpolation of the noisy blood pressure readings to a new sampling frequency f_BP_ set as once per minute.

Let the *i*^th^ BP observation, $y_i$, at time $t_i$ for a participant be modelled by the relation:

4 $$\begin{aligned} y_i = f(t_i) + \epsilon _i, \qquad i = \{1, \dots , N_{\text {meas}}\} \end{aligned}$$

where $N_{\text {meas}}$ is the number of BP measurements recorded for the participant. $\epsilon _i$ forms a sequence of independently distributed random variables with zero mean. Cubic smoothing splines define an estimate, $\hat{f}$, of *f* that equates to a cubic spline with knots (transition points) at $\hat{f}(t_i)$. At these transition point, the values of $\hat{f}$, $\hat{f'}$, and $\hat{f''}$ (where $f'$ and $f''$ denote the first and second derivative of *f* respectively) all match. The exact form of $\hat{f}$ is determined by minimising a loss $L^{\text {BP}}$:

5 $$\begin{aligned} L^{\text {BP}} = p \sum _{i=1}^{N_{\text {meas}}} (y_i - \hat{f}(t_i))^2 + \int _{t_1}^{t_{N_{\text {meas}}}} \hat{f}''(t)^2 dt \end{aligned}$$

The first term of equation (5) imposes a penalty for the squared distance between observed BP values, $y_i$, and smoothed values $\hat{f}(t_i)$. The second term imposes a penalty for the complexity of $\hat{f}$, modelled as the accumulated second derivative of $\hat{f}$. *p* is a constant that defines the relative weight placed on minimising the residual sum of squares against the complexity of $\hat{f}$. A very low value of *p* will result in the regressed function converging to a linear least squares estimate. A very high value of *p* will result in the smoothing spline converging to a cubic spline that passes through all data points.

As all participants were under the same protocol, we implemented a *p* value for SBP, MAP and DBP ($p_{\text {SBP}}$, $p_{\text {MAP}}$ and $p_{\text {DBP}}$ respectively) that was common for all of them. Each respective *p* value was determined by extending the ordinary cross-validation strategy proposed in^65^ by a grid search across the log-scaled range [$10^{-3}$,..., $10^{8}$]. For each participant, the leave-one-out (LOO) RMSE was computed across the entire *p* range. The *p* value that minimised the participant-wise average LOO error was used.

### Estimating changes in BP

We processed PPG features and PAT similarly to the methods proposed in^1^. This included: outlier detection to remove statistically significant deviations in values and a Kalman filter to reduce the effect of transient artefacts caused by noise. We then averaged the feature values within a window, *w*, of length 40s centred around each reference BP measurement (20s to the left, 20s to the right). Only beats of good quality, given by SQI_PPG_ > 0.8 and SQI_PAT_ > 0.8 respectively, were included in the window and if more than half of the window was deemed to be of bad quality, then the feature was not recorded for that window. We computed ECG features within the same window, *w*. If less than half of the window was deemed to be of good quality (SQI_ECG_ > 0.8), then feature values were not recorded for that window. We handled missing data, caused by poor signal quality, by nearest neighbour imputation for each participant.

**Figure 2:**
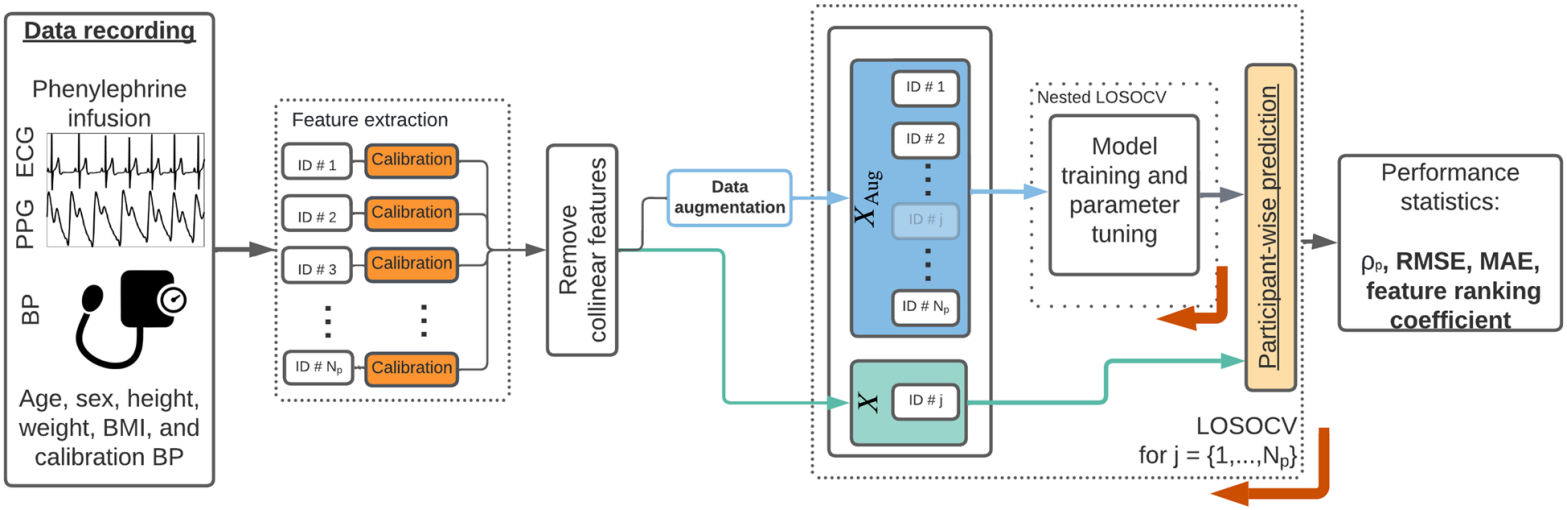
Schematic of the $\Delta$BP estimation pipeline for each of the proposed models. We extracted features from the PPG and ECG and averaged their values within a window of size 40s centred on times of cuff inflations. We then implemented a *hybrid calibration* approach such that the proposed models estimate $\Delta$BP from a baseline calibration value determined during the rest period. Data augmentation was implemented to increase the training and validation set size by interpolating between cuff inflations. Models were trained and evaluated in a nested leave-one-subject-out cross-validation (LOSOCV) framework shown here by the iterator *j* which indicates the test participant for that iteration. Participant *j* was then removed from the training/ validation set ($X_{\text {Aug}}$) for that iteration.

A schematic outlining our proposed steps for estimating $\Delta$BP is shown in Fig. 2. In this work, we adopted a *hybrid calibration*^66^ approach in order to estimate $\Delta$BP using one of two ML regression models (LASSO+OLS or RF, defined in the sections below) as a function of an input feature set, *X*. We use $x_i$ to represent the *i*^th^ observation of the feature set and $x_{ij}$ to represent the datapoint of the *i*^th^ observation of the *j*^th^ feature. We implemented four different feature sets based on the different signals being analysed in this study. For each of the following groups we restricted *X* such that it includes features only from these sources: $X \in$ {PPG, ECG, PPG+ECG, PAT}. We refer to these models and feature set combinations as LASSO + OLS_PPG_ for a LASSO + OLS model with a PPG feature set, RF_PPG + ECG_ for a RF model with PPG + ECG feature set, and so on.

We calibrated all feature and BP values to the individual participant using data recorded during the rest period of the study (first 5 minutes of the recording). We then removed all collinear features and implemented data augmentation to increase the size of the training set, *X*_Aug_. We used nested leave-one-subject-out cross-validation (LOSOCV) to train, tune and evaluate the models. For each fold of the LOSOCV, one participant in turn was set as the test participant. Data from *X*_Aug_ set for all participants apart from the test participant were used to train and tune the models. Nested LOSOCV was used for model validation to optimise model hyperparameters ($\lambda$ for OLS+LASSO and *mtry* for RF) with the aim of minimising RMSE. Data from *X* for the test participant was then used to evaluate the performance of the model. We used the following metrics to evaluate model performance: $\rho_{p}$, RMSE, and mean absolute error (MAE). Differences in performance metrics across all folds from the models were evaluated for statistical significance by a two-tailed Wilcoxon signed-rank test. We adjusted the *p*-values for multiple comparisons using the Benjamini-Hochberg method^67^. This technique aims to control the number of type I errors (incorrectly rejecting the null hypothesis) by inflating the lowest *p* values (see^67^ for more details).

#### Calibration

We adopted a *hybrid calibration*^66^ approach to personalise the estimation models for each participant in our dataset. We use the superscript *p* to reflect an observation from the *p*^th^ participant. In this work, for each participant, we defined the baseline calibration value of BP ($\widehat{\text {BP}^p}$) and the *j*^th^ waveform feature ($\widehat{f_{j}^p}$) as their respective mean values during the 5-minute resting period at the start of the study. In the specific example of one participant for whom no BP readings were taken during the rest period, we used the mean values in a one-minute window centred on the first cuff inflation as the calibration readings.

For the *p*^th^ participant, BP values were subtracted from their calibration value to compute $\Delta \hbox {BP}_i^p$, as observations of the regression target vector. Additionally, the feature set *X* consisted of relative changes of each waveform feature from their calibration value^68^. Thus $\Delta$BP and $x_{ij}^p$ took the form:

6 $$\begin{aligned} \Delta \text {BP}_i^p = \text {BP}_i^p - \widehat{\text {BP}^p}, \qquad x_{ij}^p = \frac{f_{ij}^p - \widehat{f_{j}^p}}{\widehat{f_{j}^p}}, \qquad i = \{1, \dots , N\}, \qquad j = \{i = 1, \dots , M\}, \qquad p = \{1, \dots ,N_p\}. \end{aligned}$$

where *N* and *M* are the number of observations and features in *X* respectively. $N_p$ is the number of participants.

*Hybrid calibration* strategies use participant demographics to personalise the model outputs. Thus, for all feature sets, *X*, we added participant age, sex, height, weight, BMI, and BP calibration ($\widehat{\text {BP}^p}$) as static categorical features.

#### Baseline reference

For reference performance metrics, we implemented a simple baseline reference that assumed no BP changes for each participant from their baseline calibration value $\widehat{\text {BP}^p}$ (i.e. $\Delta \hbox {BP}_i^p$ = 0 for all *i* and *p*). We refer to this as *Baseline reference* and it indicates the minimum performance that must be achieved by the regression models.

#### Removing collinear features

Collinearity occurs when there is intercorrelation between multiple features^69,70^, thus violating the independent identically distributed (i.i.d.) assumption that is common in regression models. Additionally, the presence of collinearity inflates the variance of the regression parameters and makes it difficult to assess the importance of features. Collinearity in a feature set, *X*, can be highlighted by the condition number $\kappa$ representing the ratio of the largest singular value of *X* to the smallest singular value. It can be computed as:

7 $$\begin{aligned} \kappa (X) = \left\| X^{+}\right\| \cdot \left\| X\right\| \end{aligned}$$

where $\left\| \cdot \right\|$ is the 2-norm of a matrix and $X^{+}$ is the pseudo-inverse of the matrix *X*. Typically, a condition number greater than 30 is thought to indicate the presence of strong multi-collinearity in the dataset^70^.

It is likely that collinearity exists in the feature sets presented in this paper as there are multiple features describing similar characteristics, for example entropies of the ECG or time durations of the PPG. In order to remove the effect of collinearity and to allow for parsimonious models, we removed collinear features by investigating the variance inflation factor (VIF) defined as:

8 $$\begin{aligned} \text {VIF}_j = \frac{1}{1 - R_j^2} \end{aligned}$$

where $R_j^2$ is the unadjusted coefficient of determination for regressing the $j^{\text {th}}$ feature on the remaining ones. If a feature can be accurately predicted using one, or more, other features then $\text {VIF}_j$ would be large for that feature. Removing collinear features is an iterative process where on each iteration, the feature with the largest corresponding $\text {VIF}_j$ is removed from the feature set until no features had a VIF greater than 10 (corresponding to $R_j = 0.9$)^70^.

#### Data augmentation

We implemented data augmentation in order to increase the feature set size by incorporating information from feature values between the reference BP cuff inflations. As shown in Fig. 2, we performed model training and validation on the augmented feature set, hereafter referred to as *X*_Aug_, and performance metrics were reported using the original dataset *X*. We constructed *X*_Aug_ by interpolating between the reference BP values for each participant using the cubic smoothing splines outlined above at a new frequency, *f*_BP_ = 1/15 Hz (four measurements per minute, as opposed to once a minute) with a smaller window size, *w* = 15s, to prevent overlapping windows violating the i.i.d assumption.

#### Regression models

The models we implemented to estimate $\Delta$BP are outlined below. Different models were built for estimating $\Delta$SBP, $\Delta$MAP and $\Delta$DBP.

**LASSO + OLS** To explore the linear relationship between each of the feature sets *X* and $\Delta$BP, ordinary least squares (OLS) linear regression was implemented. In order to prevent over-fitting and to improve model interpretability, we employed the Least Absolute Shrinkage and Selection Operator (LASSO) method to remove redundant features prior to linear regression. We refer to this model as LASSO+OLS. LASSO imposes the L1-norm penalty to the residual sum of squares using non-negative values of shrinkage parameter $\lambda$. LASSO allows the removal of features by shrinking some feature coefficients, $\beta$, towards zero:

9 $$\begin{aligned} \beta = \mathop {\textrm{argmin}}\limits _{\beta } \Bigg(\sum _{i=1}^{N}(Y_i - x_i \beta )^2 + \lambda \sum _{j=1}^{M}|\beta _j|\Bigg) \end{aligned}$$

where *Y* is the target vector ($\Delta$BP values) of length *N*, and *M* is the number of features. We optimised the $\lambda$ hyper-parameter by a nested LOSOCV loop. For each loop of the LOSOCV, LASSO feature selection was implemented and features with non-zero coefficients were used by OLS to compute $\Delta$BP estimates.

**Random forest** To explore potentially non-linear relationships between *X* and $\Delta$BP, we additionally built a Random Forest (RF) regression model. RF regression models utilise majority voting across multiple decision trees, each trained with a split criterion based on summed squared error (SSE)^71^. Each decision tree in an RF model was trained on a bootstrap of features. This approach reduces model variance whilst maintaining a low bias. As RF models select features upon training, we trained the model using all available features (i.e. without the need for LASSO). Typically, RF models are not very sensitive to choices in the number of trees (*N*_trees_), provided it is sufficiently high^71^. Therefore, we set the number of trees to 300. We optimised the number of features randomly selected for each node (labeled *mtry*) by a nested LOSOCV loop.

#### SHAP values feature ranking coefficient

A key objective of this work was to highlight features that have strong predictive power for estimating $\Delta$BP. We assessed the importance of each feature through Shapley additive explanation (SHAP) values^72^. Shapley values, $\phi _j$, represent the marginal contribution of each feature to individual model predictions. For a given regression model $f(\cdot )$, the marginal contribution of the *j*^th^ feature is computed through the difference in model outputs when trained with that feature compared to when trained without it, $f(z_i \cup \{x_{ij}\}) - f(z_i)$, where $z_i$ is a subset of features in $x_i$ not including $x_{ij}$ ($z_i \subseteq x_i \setminus \{x_{ij}\}$). Since the impact of adding a feature depends on the other features in the model, $\phi _j$ is computed as the weighted sum of marginal contributions of the *j*^th^ feature over all possible subsets $z_i \subseteq \{x_{i1}, \dots x_{iM}\} \setminus \{x_{ij}\}$:

10 $$\begin{aligned} \phi _j(f, x_i) = \sum _{z_i \subseteq x_i \setminus \{x_{ij}\} } \frac{|z_i|!(M - |z_i| - 1)!}{M!}(f(z_i \cup \{x_{ij}\}) - f(z_i)) \end{aligned}$$

where $|z_i|$ is the number of features in $z_i$. As most models cannot handle missing data, SHAP values are computed by replacing $f(\cdot )$ in equation (10) with $f_x(\cdot )$, a conditional expectation function of the original model. This step equates to replacing each missing feature with a random value representative of the datapoints the original model was trained over. Repeating this process, and averaging the results integrates out the missing value. It is demonstrated in^72^ that SHAP values computed in this manner correspond to the only method of assessing feature importance that satisfies three desirable properties known as local accuracy, missingness, and consistency. A result of these properties is that for a complex, non-linear, model such as RF, a simpler explanation model $g(\cdot )$ can be approximated through the linear combination of SHAP values across all features:

11 $$\begin{aligned} g(x_i) = \phi _0 + \sum _{j = 1}^{M}\phi _j \end{aligned}$$

where $\phi _0$ is the expected model value over the training set. Equation (11) allows for local interpretability of model outputs by examining each feature’s SHAP value for a given estimation. As SHAP values are computationally expensive to compute (there are $2^M$ distinct coalitions of feature values), we computed approximates for the SHAP values through KernelSHAP (for LASSO+OLS) and TreeSHAP (for RF) implementations in the SHAP Python library^72,73^.

For each loop of the cross-validation (CV), we assessed the overall importance of each feature as the mean absolute SHAP value across the training data. To report the variability of the feature importance across the CV , we computed a ranking coefficient. The ranks of all features were determined at each fold and normalised by the total number of features (1 being the highest rank, 0 being the lowest). For each feature, the distributions of the ranking coefficients across all folds were analysed. This step allowed for each fold of the LOSOCV to contribute equally to assessing the overall importance, while also providing a fair comparison across SBP, MAP, and DBP.

## Results

### Clinical study

Thirty volunteers were recruited for our clinical study. We discarded the data from four participants from the analysis. For three of these participants, the reference ECG waveform did not include any periods of high-quality data as a result of errors in the connection of the ECG electrodes. For one participant there were errors recording the BP cuff data. Therefore, 26 participants made up our dataset. The demographics of the participants in the study whose data was used for analysis are shown in table 4. All participants were healthy with a median BMI of 22.5 kg/$\hbox {m}^{2}$ and no history of cardiovascular disease. The median age of participants was 28 years and there was an even split of sexes (53.8% female). On average, we achieved an increase of 20 mmHg in SBP, with a maximum increase of 40 mmHg in a subset of participants.

**Table 4:** Demographics of the population in the clinical study.

Descriptor	Value
Total number of participants	26
Male	12
Female	14
Average length of session (mins)^1^	28.0 (0.1)
Age (years)^1^	28.0 (9.0)
Height (cm)^1^	170.0 (18.0)
Weight (kg)^1^	69.5 (23.0)
Body Mass Index (kg/$\hbox {m}^{2}$)^1^	22.5 (5.2)
Reference maximum $\Delta$ BP per participant^1^	
$\Delta$ SBP (mmHg)	20.0 (8.0)
$\Delta$ MAP (mmHg)	17.0 (10.0)
$\Delta$ DBP (mmHg)	15.5 (9.0)
Reference $\Delta$ BP values^2^	
$\Delta$ SBP (mmHg)	6.5 (10.4)
$\Delta$ MAP (mmHg)	5.3 (8.8)
$\Delta$ DBP (mmHg)	4.7 (8.2)

^1^ Participant-wise median (IQR), ^2^ mean (standard deviation) across dataset.

### Removing collinear features

**Figure 3:**
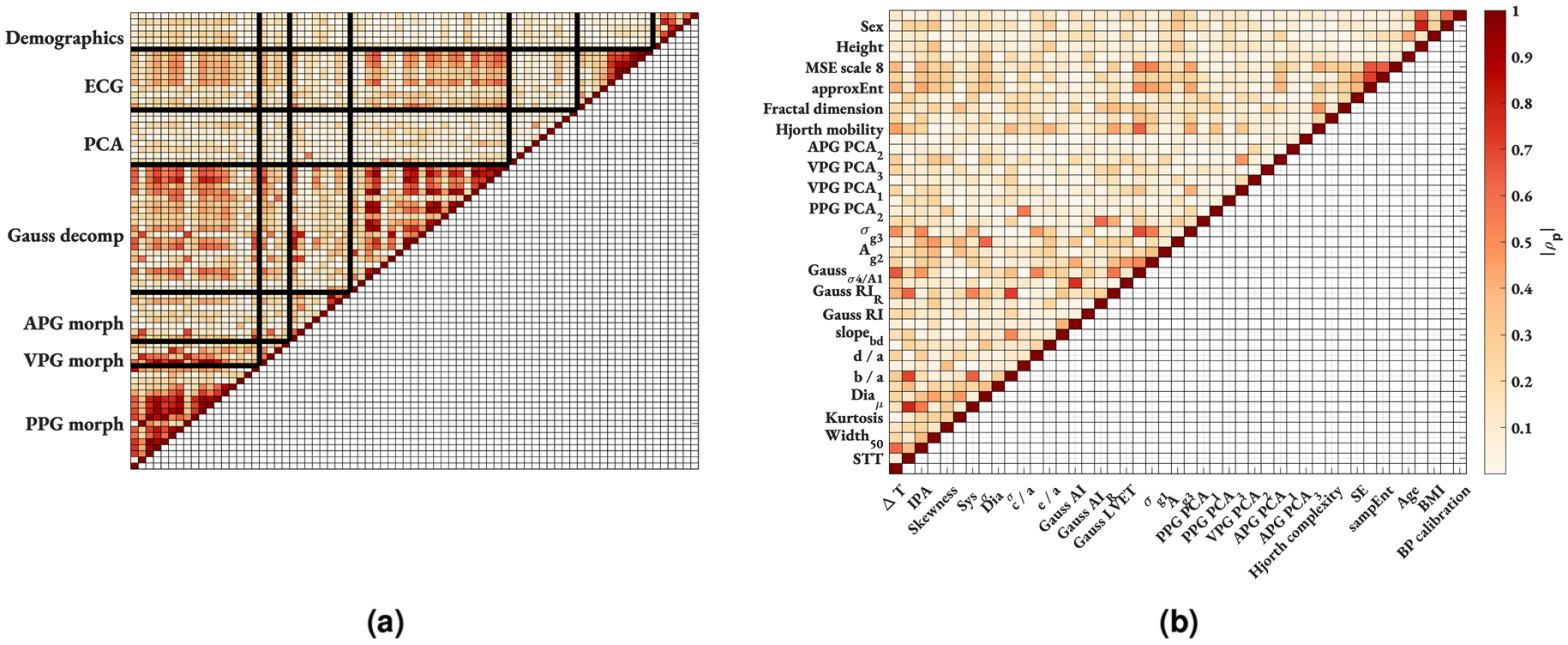
Results of removing collinear features in PPG + ECG feature set. Pairwise linear absolute Pearson’s $|\rho _p|$ correlation matrix. (**a**) pre and (**b**) post removal of collinear features. Blank spaces represent non-significant ($p > 0.05$) or weak ($|\rho _p| < 0.2$) correlations. For clarity of labelling on the post-collinear feature set (**b**), every other label is presented on the y-axis with the remaining labels on the x-axis.

Figure 3a shows the correlation matrix of the total feature set (PPG + ECG + demographics), including 77 features. There were a large number of features (58.5%) that were significantly correlated ($|\rho _p| >0.8, p < 0.05$) with at least one other feature. This indicates a high level of collinearity, highlighted by a condition number $\kappa$ of 315. Figure 3b shows the correlation matrix after removing all collinear features. The remaining dataset contained 45 features with a condition number $\kappa$ of 11, suggesting independence of features and encouraging parsimonious models. For completeness, Supplementary Information table SI 3 provides a list of the remaining features and the subset of the total feature set with which they have a strong correlation. Additionally, Supplementary Information table SI 3 provides the correlation with $\Delta$SBP for each feature across the whole cohort and on a participant-wise basis.

### Comparing model performance

Table 5 shows the median and interquartile range (IQR) performance statistics for all models computed over all 26 folds of the LOSOCV for $\Delta$SBP. The results for $\Delta$MAP and $\Delta$DBP are provided in the Supplementary Information table SI 1 and table SI 2 respectively. We note large RMSE and MAE values for the *baseline reference* indicating that participants experienced a significant change in their SBP values in response to the weight-based dosing of phenylephrine.

All models outperformed results obtained with the *baseline reference*, and all apart from LASSO + OLS_ECG_ reported statistically significant *p* values indicating that consistent improvements in performance statistics were observed ($p < 0.05$ for all). For the PPG, ECG and PPG+ECG feature sets, the RF model consistently achieved stronger performance metrics than LASSO + OLS. Statistically significant improvements were recorded only in RMSE and MAE ($p < 0.05$ for all). The Wilcoxon signed rank test failed to reject the null hypothesis of equal median $\rho _p$ between LASSO+OLS and RF. The PPG feature set significantly outperformed the ECG feature set for all performance metrics and regression models. The absolute difference in the median $\rho _p$, RMSE, and MAE between RF_PPG_ and RF_ECG_ was 0.19 ($p = 0.00007$), 1.04 mmHg ($p = 0.005$) and 0.53 mmHg ($p = 0.004$) respectively. RF_PPG_ and RF_PPG + ECG_ reported similar performance statistics with non-significant *p* values, indicating that adding ECG features to a feature set of PPG features offers little or no performance gain. For PAT, LASSO + OLS significantly outperformed RF across all performance metrics ($p < 0.05$ for all). LASSO + OLS_PAT_ achieved similar performance metrics to the RF_PPG + ECG_. LASSO + OLS_PAT_ consistently resulted in the smallest IQR for all performance metrics.

**Figure 4:**
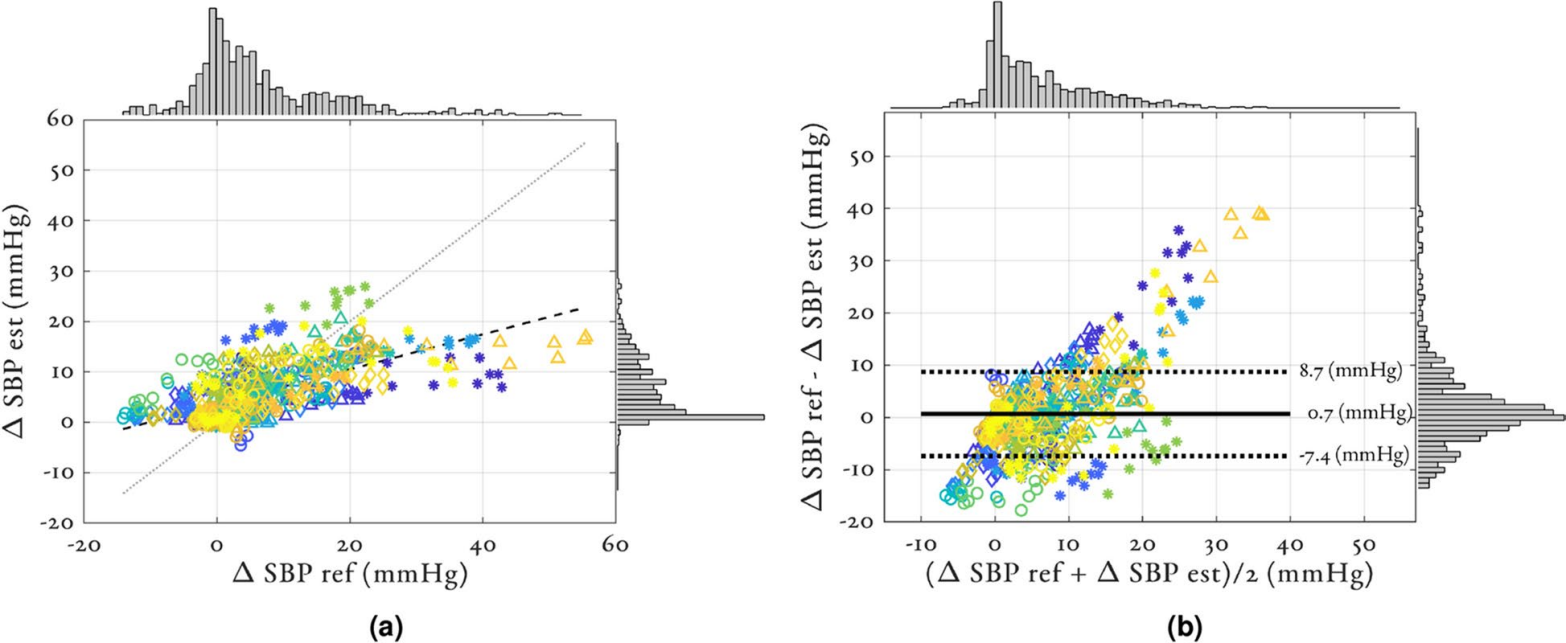
Agreement between the reference $\Delta$SBP values from the sphygmomanometer cuff and the estimated $\Delta$SBP using RF_PPG + ECG_. Individual participants are colour and marker-coded. (**a**) The correlation analysis, the overall correlation was 0.64, the median participant-wise correlation coefficient was 0.86 with a range of 0.34 to 0.95. Black striped line shows regression line. Grey dotted line shows line of unity. (**b**) The Bland-Altman analysis, the bias of the overall error was 0.3 mmHg with a standard deviation of 8.05 mmHg.

Figure 4 shows the (a) correlation and (b) Bland-Altman plots for $\Delta$SBP estimation using the RF_PPG + ECG_ model. Supplementary Information figures SI 2-3 show the correlation and Bland-Altman plots for $\Delta$MAP and $\Delta$DBP respectively. Individual participants are colour and marker-coded. The $\rho _p$ value of the overall estimation was 0.64. The median participant-wise correlation coefficient was 0.86 with a range of 0.34 to 0.95. Figure 4a shows the histograms of the reference and estimated values. The reference $\Delta$SBP values ranged from -16.4 to 53.8 mmHg, but the estimated $\Delta$SBP values had a much tighter range of -3.37 to 22.2 mmHg. The bias of the overall error was 0.30 mmHg with a standard deviation of 8.05 mmHg (see Fig. 4b). We note also an additional bias where large values of $\Delta$SBP were underestimated and small values were overestimated. At peak infusion, the median (IQR) value of $\Delta$SBP across the cohort was 20 (8) mmHg (see table 4). We found large errors in the data for the four participants whose $\Delta$SBP at peak infusion exceeded 30 mmHg.

Supplementary Information figures SI 5-7 show the individual reference $\Delta$BP and estimated $\Delta$BP values using the RF_PPG + ECG_ model across all participants in the study for SBP, MAP and DBP respectively.

**Table 5:** Performance statistics of $\Delta$SBP estimation using the models proposed. Results are given as median (IQR) computed across all folds of the LOSOCV. Entries in bold indicate the best performance for that metric.

Model Name	$\rho _{P}$	RMSE *	MAE *
*Baseline reference*	- (-)	10.46 (6.18)	7.40 (4.87)
LASSO+$\hbox {OLS}_{\rm{PPG}}$ $\hbox {RF}_{\rm{PPG}}$	0.85 (0.44) **0.88 (0.26)**	6.19 (4.05) 5.75 (4.73)	5.41 (2.41) **4.75 (3.66)**
LASSO+$\hbox {OLS}_{\rm{ECG}}$ $\hbox {RF}_{\rm{ECG}}$	0.64 (0.70) 0.69 (0.45)	7.48 (6.37) 6.79 (5.66)	6.54 (4.63) 5.28 (4.57)
$\hbox {LASSO+OLS}_{\rm{PPG+ECG}}$ $\hbox {RF}_{\rm{PPG+ECG}}$	0.82 (0.40) 0.86 (0.23)	7.99 (5.74) **5.66 (4.76)**	6.30 (4.62) 4.86 (4.29)
$\hbox {LASSO+OLS}_{\rm{PAT}}$ $\hbox {RF}_{\rm{PAT}}$	0.83 (0.10) 0.80 (0.15)	6.09 (4.25) 6.39 (4.23)	4.91 (3.33) 5.21 (3.69)

* results given in units of mmHg.

**Figure 5:**
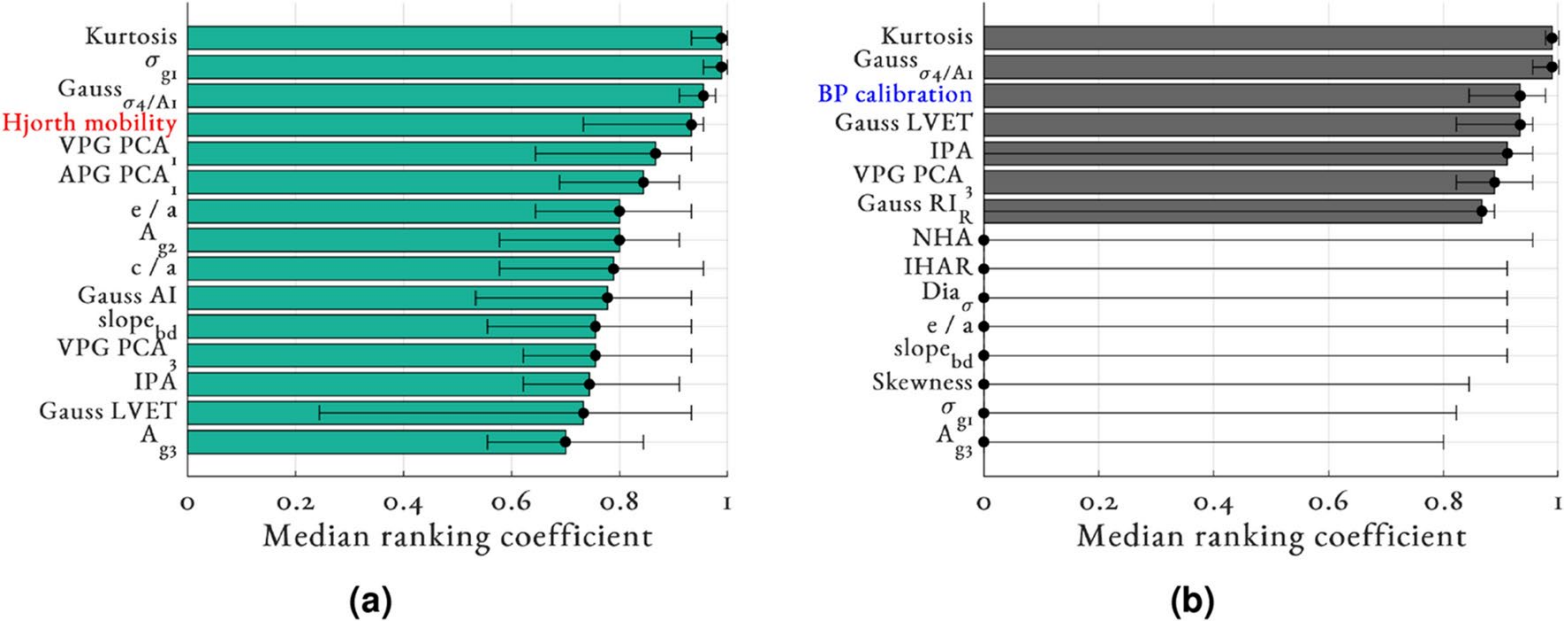
Median SHAP values feature ranking coefficient for (**a**) random forest and (**b**) LASSO+OLS both with PPG + ECG feature set. Features are ordered by their respective median ranking coefficient. Error bars denote the range. Demographic features are highlighted in blue and ECG features in red for clarity. For brevity, only the top 15 features are provided here with the full list provided in Supplementary Information figure SI 4.

### Feature importance

To mitigate the variations in the training data between folds of the LOSOCV, overall SHAP value feature importances was assessed using a ranking coefficient. Figure 5 shows the median (across folds) RF and LASSO+OLS ranking coefficients for the PPG+ECG feature set for predicting SBP, with only the top 15 features shown. In Supplementary Information figure SI 4, we show all features’ median ranking coefficients for estimating SBP, MAP, and DBP. We quantified the agreement between the feature ranks for pairs of SBP, MAP, and DBP using the Kendall rank correlation coefficient, $\rho _k$^74^. It was found that the feature importance for SBP, MAP, and DBP estimation showed strong agreement with each other ($\rho _k > 0.6$ for all) and as a result we continue to report SBP in the main body of this work. The top five features as determined by the median RF ranking coefficient were: kurtosis, $\sigma _{g1}$, $\hbox {Gauss}_{\sigma 4/A1}$, Hjorth mobility, and VPG $\hbox {PCA}_1$. The top five features as determined by the median LASSO+OLS ranking coefficients were: kurtosis, $\hbox {Gauss}_{\sigma 4/A1}$, Gauss LVET, BP calibration, and IPA. Kurtosis had the highest feature importance in 13 out of 26 folds for both RF and LASSO + OLS (2^nd^ highest in the remaining folds). In general, the ranking of features by LASSO+OLS followed the order of feature correlation values shown in Supplementary Information table SI 3. The only ECG feature that demonstrated significant importance in $\Delta$BP estimation was Hjorth mobility which had a median ranking coefficient of 0.93 for RF.

**Figure 6:**
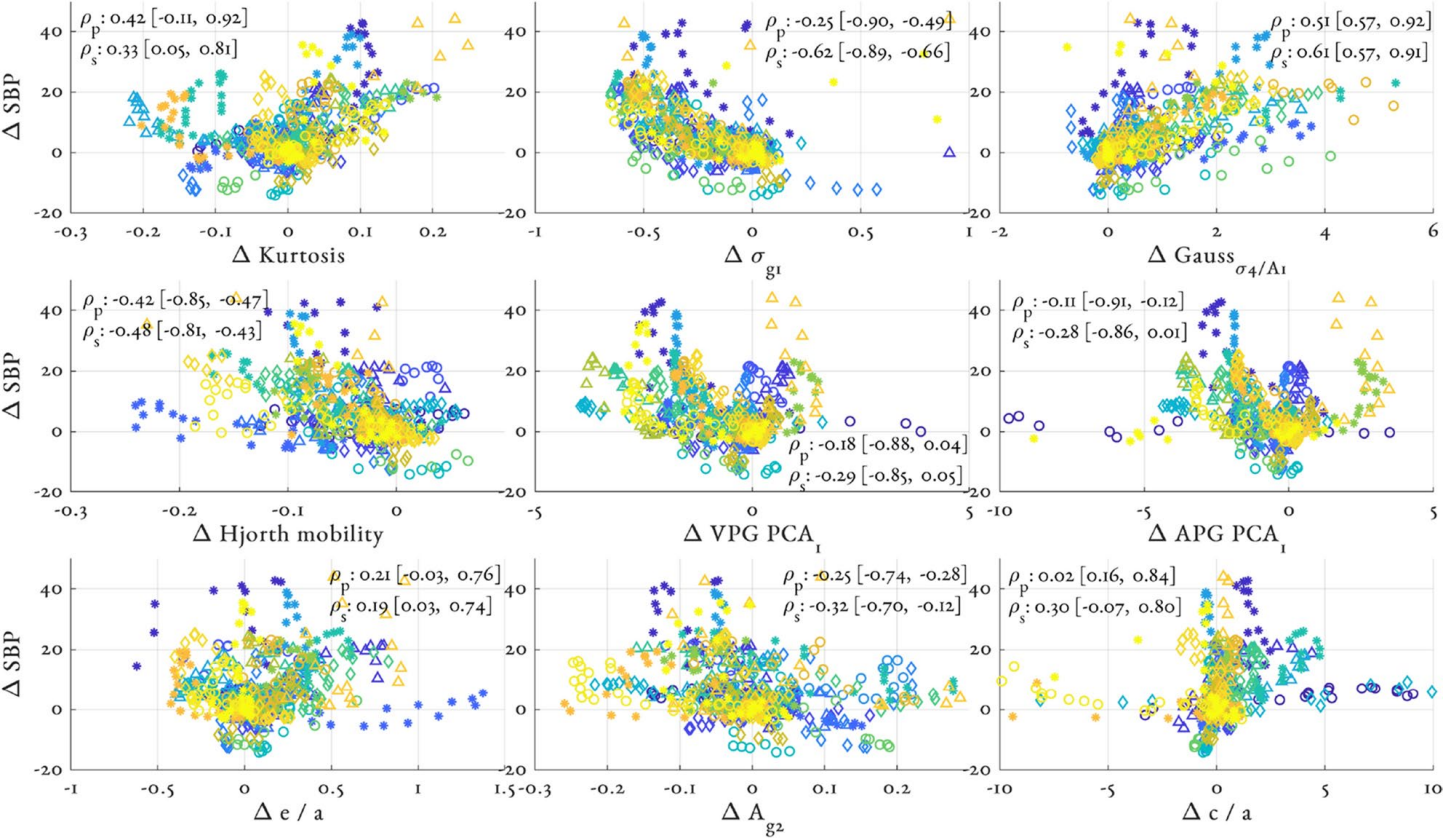
Relationship between top 9 ranking, non-demographic, features from the RF_PPG+ECG_ model and $\Delta$SBP. Individual participants are colour and marker coded. Inter Pearson’s $\rho _p$ and Spearman’s $\rho _s$ correlation coefficient are shown as median and upper and lower quartiles. Additionally the 1st and 3rd quartiles of the participant-wise correlation coefficients are shown. Note that feature values have been both subtracted and divided by their value at baseline.

Figure 6 shows the relationship between $\Delta$SBP and the top 9 ranking, non-demographic, features from the RF_PPG + ECG_ model. Different participants are coded by colour and marker to highlight clusters of features suggesting individual-specific feature changes. Both Pearson’s $\rho _p$ and Spearman’s $\rho _s$ correlation coefficient are provided. Spearman’s correlation indicates monotonic (but not necessarily linear) relationships and so may provide further insight into the RF models (see for example the scatter of $\Delta \sigma {g1}$).

## Discussion

This work describes the methods for the non-invasive estimation of $\Delta$BP in healthy participants using morphological features from the PPG and ECG waveforms. Changes in BP were induced by the infusion of phenylephrine using a standard weight-based dosing protocol, instead of being BP-target driven (although BP was constantly under review by the clinician to ensure safety of the participants). One of the key advantages of this study was that, in a relatively short period of time, and while remaining supine and still, the participants experienced a wide range of BP values. This helped to validate algorithms for non-invasive, cuffless, estimation of SBP over a clinically useful range of $\Delta$SBP (-10 to 30 mmHg).

The PPG and the ECG waveforms offer great potential for non-invasive monitoring due to their ubiquity and ease of acquisition. The PPG in particular can be acquired using wearable devices such as smartwatches or using video plethysmography^14^. BP estimation using the PPG has been studied in a number of papers^14^, however the best features and models have remained unclear. A single-lead ECG may be recorded by three electrodes or via capacitive coupling^75^. The relationship between changes in BP and the ECG is governed by mechano-electric coupling (MEC). However, this connection is considered less robust to that of the relationship between the PPG waveform and BP. As a result, estimating $\Delta$BP from the ECG waveform has been explored in less detail in the literature.

### Observed changes in the PPG waveform morphology

Figure 1 shows the changes in PPG beat seen for a typical participant during the four main stages of our study (a) rest, (b) dose increase, (c) max infusion, and (d) washout. This includes a loss of a clear dicrotic notch and a rising middle peak (often referred to as a tidal wave^9^ and thought to be caused by reflected waves at the renal arteries^8^) that can overshadow the initial peak seen at rest. These changes have been reported previously^18,41,49^ and are thought to be due to increasing amplitude and speed of reflected waves due to arterial stiffening. Additionally, they have also been shown to occur for age related arterial stiffening^9^. Note that in Fig. 1c at maximum infusion, the tidal wave has a peak greater than that of the original systolic peak (see Fig. 1b). As the systolic peak is almost universally defined as the maximum of the PPG beat^37^, this may lead to the tidal wave being incorrectly classified as the systolic peak. In which case, features such as CT, $\Delta$T, and STT, which rely on accurate systolic peak detection would be significantly affected. To account for this, we detected the systolic peak as the first turning point of the PPG pulse above its midpoint, as can be seen in Fig. 1c. We found that this definition was acceptable for our short, single perturbation study with minimal motion artefacts.

Other changes observed in the PPG waveform morphology during the phenylephrine infusion include variations in amplitude and time duration. Many factors can impact the amplitude of the PPG pulse, for example: respiration^33^; changes in the contact pressure of the pulse oximeter^76^; and changes in peripheral blood volume^6^. As these factors can occur independently of changes in blood pressure, and as recommended in^76^, we normalised the PPG pulse to have unit amplitude. Additionally, phenylephrine infusion activates the baroreflex response, increasing the parasympathetic nerve activity to the heart, and slowing heart rate down^77^. It was therefore decided to additionally normalise the time duration of the PPG pulse in order for features such as $\Delta$T (the time delay from systolic peak to dicrotic notch) to be independent of HR.

### Model performance

Typically, machine learning algorithms require a large amount of data in order to make robust estimations. In this work, we were limited by the number of participants in the study ($N_p$=26) and by the number of data points per participant (typically once per minute for the 28 minutes of the recording per participant). In order to overcome these limitations, the models were trained, tuned and evaluated with a LOSOCV framework. Additionally, we implemented data augmentation to increase the number of data points in our training set by interpolating between the cuff measurements using cubic smoothing splines with an additional 3 augmented measurements every minute. Across all models, this data augmentation statistically significantly improved the performance statistics at the $p < 0.001$ level, computed using a Wilcoxon signed rank test. Figure 3a splits all features into groups (PPG morphology, VPG morphology, APG morphology, Gaussian decomposition, ECG and demographics) and shows strong intra-group correlations indicating multi-collinearity in the dataset. We reduced feature collinearity by removing features with a VIF $> 10$. This increased the confidence of the model parameters, allowed for an appropriate examination of feature importance and encouraged parsimonious models.

For the PPG, ECG, and PPG+ECG feature sets, there were statistically significant improvements in RMSE and MAE when using the RF model compared to LASSO+OLS model at the $p< 0.05$ level as computed by the corrected Wilcoxon signed rank test, whereas a non-significant difference was found in $\rho _p$. This indicates that LASSO+OLS was able to appropriately detect directional changes in $\Delta$SBP, although it was unable to determine the magnitudes of the changes. This points to a calibration issue whereby the LASSO+OLS model was unable to approximate the feature calibration gradients for each participant. RF models may have been better at estimating the feature calibration gradients as these models allow for interactions between features and demographics. Indeed, larger overall importance was placed on demographic features by RF_PPG+ECG_ than LASSO+OLS_PPG+ECG_ (see Supplementary material figure SI 4). In linear models these static features can only influence intercepts and not gradients. Thus since the feature set was calibrated in order to predict $\Delta$BP from a baseline calibration value, low overall importance was placed on demographics by LASSO+OLS_PPG+ECG_, with the exception of BP calibration. Supplementary material figures SI 5-7 demonstrates the marginal influence of demographics on the RF_PPG+ECG_ model estimates through the use of SHAP values. Demographics were found to have a varying influence on the $\Delta$BP estimation, with a positive impact on some participants (IDs: 003, 009 for example) and a negative impact on others (IDs: 018, 025 for example). Demographics are known to influence the PPG contour; in particular, c/a has been previously explored as a marker for age-related arterial stiffening^41^. Interestingly, the RF_PPG+ECG_ model puts little to no emphasis on the sex of the participant (see Supplementary Information figure SI 4) despite there being evidence of a sex-related dependency on the PPG morphology^16^. We would recommend in future studies that the interactions between participant demographics may be accounted for in a linear model by implementing a linear mixed-effects model with random effects for parameters such as age and BMI. We were unable to explore this line of work here due to the limited number of participants and their relative homogeneity.

Despite interactions with demographics, a significant calibration issue still persisted. Figure 4 shows the results of $\Delta$SBP estimation using the RF_PPG + ECG_ model via correlation and Bland-Altman analysis. Xing et al.^78^ reported a Bland-Altman plot with similar characteristics where the model estimated $\Delta$SBP values to be in a much tighter range than was given by the reference and there were large errors found particularly at the high values. The largest errors found in all models occurred in the four individuals with the largest $\Delta$SBP at peak infusion (IDs: 002, 010, 023 and 026 in Supplementary Information figure SI 5). These four individuals experienced significantly larger values of $\Delta$SBP at peak infusion than the rest of the cohort ($\Delta \hbox {SBP} > 33$ mmHg, whereas the median value across the cohort at peak infusion was 20 mmHg). The precise clinical effect of the weight-based dosing of phenylephrine in an individual would depend on the balance between their sensitivity to the increase in afterload, the effect of bradycardia on cardiac filling (therefore contractility, the Frank-Starling law^79^), and the proportion of venous/arterial action of phenylephrine causing the increase in preload and afterload. As a result, variations in $\Delta$BP at peak infusion were expected across the cohort. The median weight of the four outlier individuals (73 kg) was marginally larger than the cohort average (69.5 kg) and so on average a larger dose of phenylephrine would have been given. Other post-hoc assessments of demographics are not sufficient to distinguish these four individuals. Although in such a small cohort it is difficult to draw conclusions for these individuals, we suggest two possible explanations for the large errors found. Firstly, these individuals clearly experienced a significant change to their cardiovascular system in response to the dosing of phenylephrine and so the changes in the cardiovascular system may not be adequately represented by the features available. Secondly, the *hybrid calibration* strategy may be impacted by the small sample size. As a result, there will be difficulty in calibrating individuals that may be classed as outliers. In further work, to combat this inadequate calibration we suggest gathering a more longitudinal dataset for each individual and implementing *individual calibration* strategies.

Likely driven by the linear relationship between PAT and BP^1^, LASSO + OLS_PAT_ had stronger performance metrics than RF_PAT_. PAT has been investigated as a surrogate measure of BP in this dataset previously^1^ when we reported that *individual calibration*, as opposed to population-based *hybrid calibration* models, were needed for appropriate estimation of BP. In this work, there were insignificant differences found between RF_PPG+ECG_ and LASSO + OLS_PAT_ suggesting that features from the PPG and ECG have the same calibration constraint as PAT. Estimating BP from the ECG and/or PPG may hold two significant advantages over PAT. Firstly, the devices do not need to be perfectly synchronous and recorded on the same internal clock as is required for computing PAT estimates and has been reported as a limitation in certain datasets^80^. It would be possible to design a system for which BP was estimated from both devices when they are available and only from one device when the other was disconnected for any reason. Secondly, BP estimation from the PPG and ECG features was not impacted by the pre-ejection period (PEP) which is a significant limitation to BP estimation using PAT^2^. In our previous publication, we additionally reported on the influence of the pre-ejection period (PEP) on PAT estimates, noting that in this dataset PEP was found to have contributed between 28.8% and 35.2% of PAT^1^.

The results in table 5 suggest that PPG features have a significantly stronger relationship to changes in BP than the complexity features we extracted from the ECG. Additionally, despite Hjorth mobility demonstrating significant importance (median ranking coefficient = 0.93), when adding the ECG to a PPG feature set, no performance improvements were observed. Supplementary material figures SI 5-7 show the accumulated SHAP values for PPG features, ECG features, and demographics on the RF_PPG+ECG_ model estimates demonstrating that the model is largely dominated by the PPG component. As mentioned in the introduction, the theoretical relationship between changes in BP and the ECG is governed by MEC and the poor performance suggests that external, non-cardiac, control mechanisms may have a significant impact on the ECG-BP relationship, affecting the latter’s ability to estimate $\Delta$BP. An additional explanation for the poor performance of the ECG feature set may be the choice of features used to explain the changes in ECG morphology; however to the authors’ knowledge, there is no other work suggesting alternatives to ECG complexity features for BP estimation. For RF_ECG_, improvements from a naive *baseline reference* assuming constant BP values were observed, but in general we suggest that ECG features on their own may not offer a viable solution to cuffless BP monitoring.

### Feature importance

A key contribution of our work to the field of cuffless BP monitoring is a robust assessment of feature importance through the use of SHAP values and a ranking coefficient. We explored a large and comprehensive pool of features from both the PPG and the ECG gathered from a wide range of previous work (see table 3 and 2). Supplementary Information table SI 3 shows the features remaining for analysis after removing collinear features and the features from the original set with which they best correlate (defined as $|\rho _p| > 0.8, p < 0.05$). The overall correlations of these features to $\Delta$SBP across the cohort were in general quite low with only one feature ($\hbox {Gauss}_{\sigma 4/A1}$) having $|\rho _{p\Delta {SBP}}| > 0.5$. There were a number of features that had significant participant-wise correlations (PWC) to $\Delta$SBP, 21 features with a median absolute PWC $> 0.5$. The disparity between low correlations across the cohort and high correlations on a participant-wise basis underpins the important need for *individual calibration* due to low intra-participant variability and high inter-participant variability.

BP is determined by CO and TPR, and changes in either of these may be represented by different features^44^. Phenylephrine causes a direct increase in TPR^81^ via an increase in both arterial and venous vasoconstriction. Therefore, a large impact from the reflected waves, caused by impedance mismatches at points along the arterial tree (specifically the renal and iliac arteries^8^), was not unexpected. This was reflected in the observation that the majority of features that in this study have either a strong correlation or importance in estimating $\Delta$SBP, characterise the impact of the reflected pressure waves ($\hbox {Gauss}_{\sigma 4/A1}$, c/a, IPA). On the other hand, phenylephrine causes a mixed response in CO with the relationship governed by preload dependency^81^. The majority of participants in the study experienced a decrease in CO (see Supplementary Information figure SI 8), driven largely by a decrease in heart rate. Therefore, it is not surprising that at least one of the most important features ($\sigma _{g1}$) represents changes in the upslope of the PPG which is driven by changes in CO^44^. However, it is not always clear how to link one feature to a specific BP control mechanism. Kurtosis (the feature with the highest importance for both RF and LASSO+OLS) for example, represents changes in the overall shape of the PPG.

We found some agreement between the features of the highest importance in our models to those reported in the literature. For example, features derived from the APG have been previously demonstrated to reflect age-related arterial stiffening^42,46^. In agreement with this, in our work, *e*/*a*, *c*/*a*, and $\hbox {slope}_{bd}$ were found to have median ranking coefficients of 0.89, 0.84, and 0.75 respectively. In general, however, we suggest that there is little consensus on the appropriate features for BP estimation, both in our work and across the literature. There were large variations in the feature importances observed between folds (see Fig. 5). Additionally features such as STT, proposed by Addison et al.^50^ and supported by a recent publication by Natarajan et al.^68^, had a very low median ranking coefficient of 0.16 in our dataset. Similarly, the best-performing features from Sun et al.^18^ ($\hbox {Sys}_\mu$, $\hbox {Sys}_\sigma$, $\hbox {Dia}_\mu$, and $\hbox {Dia}_\sigma$) and Miao et al.^19^ (*b*/*a* and RI) all demonstrated poor performance in this work. Furthermore, to our knowledge, no previous works have suggested that Kurtosis may be a feature of significant importance for BP estimation.

A secondary explanation for the improved performance of the RF model relative to the LASSO+OLS model, may be that the features presented in this study (or at least the high-performing features presented in Fig. 5a) have a non-linear relationship to $\Delta$BP. This is further corroborated by the relationship between the top 9 ranking (non-demographic) features and $\Delta$SBP shown in Fig. 6. Only $\sigma _{g1}$ shows a discernible global relationship to $\Delta$SBP. Individual, participant-specific clusters are apparent, highlighting the low intra-participant variability but high inter-participant variability. We additionally note that even within the clusters, non-linear relationships are often observed. This non-linear relationship is additionally supported by the results of Radha et al.^12^ and Hasanzadeh et al.^22^ who both reported performance improvements when estimating BP using a RF compared to a linear model. This significantly impacts the ability to develop *individual calibration* models using these features. For PAT, typically only 2 model parameters (a slope and an intercept) are required for accurate *individual calibration*. Therefore, theoretically, a dataset containing only two measurements of PAT and BP is required for accurate calibration to an individual (although in practice this number is much higher for accurate estimation of the model parameters, see^1^). Whereas, for non-linear modelling of the PPG or ECG features, many more parameters are required to be estimated thus forcing a much larger dataset requirement for accurate *individual calibration*.

It should be noted that many of the features used in this study were derived from fiducial points such as the dicrotic notch. Relying on fiducial point detection has a number of limitations for BP estimation. The detection algorithms often set arbitrary decisions or thresholds for fiducial point locations. As discussed previously, the typical definition for the systolic peak was not appropriate in this study due to the increasing influence of the tidal wave. Additionally, fiducial point detection algorithms will be valid up to a precision; small changes in BP observed in, for example, an ambulatory setting may result in very small perturbations in feature values that are indistinguishable from errors in fiducial point detection^22^. Finally, the fiducial points are not always detectable. The dicrotic notch has been reported to diminish in elderly individuals due to atherosclerosis (hardening of vessel walls and recruitment of collagen fibres to support walls)^46,82^. We found that the majority of the features of high importance (for example: kurtosis, PCA features and Gaussian decomposition features) did not require fiducial point detection. For the reasons stated above, these may offer more desirable representation of changes of the PPG.

### Limitations

There are several limitations to the work presented in this study. Firstly, we perturbed BP via an infusion of phenylephrine, an $\alpha _1$-adrenergic receptor agonist that induces arterial and venous smooth muscle contraction^30^. In daily life, BP changes result from a diverse set of physiological mechanisms governed by the autonomic nervous system. $\alpha _1$ receptors are typically activated in response to shock or low blood pressure^83^. Whereas, the resulting smooth muscle contraction is often activated during daily life activities, such as exercise^84^, in order to ensure adequate blood flow. Further work is required to understand how the relationship observed in this work compares to that present in daily life, however data acquired during daily life will be corrupted by motion artefacts.

Secondly, our results were reported across a small number of, relatively homogeneous, healthy participants ($N_p$=26). We employed a *hybrid calibration* strategy to estimate changes in BP and utilised information from all available participants via a LOSOCV framework. However, this data-driven strategy requires a more heterogenous cohort in order to improve model accuracy. This was particularly highlighted in the calibration issue for the four individuals with the largest errors (see Supplementary Information figure SI 5). In particular, the cohort should contain participants across a wide range of age groups in order to account for variations in age-related arterial stiffness.

Thirdly, despite participants being administered a significant dose of phenylephrine (2mcg/kg/min), the changes we observed in the PPG (see Fig. 1) were very subtle. We were able to detect the fiducial points of the PPG accurately, as motion artefacts were reduced and the contact pressure of the pulse oximeter was maintained constant. However, in a real-world setting where such large variations in BP are uncommon and motion artefacts are a significant source of noise for PPG, this may be a significant limitation to BP estimation using PPG.

Finally, measurements of BP using a sphygmomanometer cuff are susceptible to various forms of noise that can distort the readings. The oscillometric device used as a BP reference in this study was compliant with the IEC 60601-2-30/EN60601-2-30 and with the American National Standard for Electronic or Automated Sphygmomanometers (ANSI/AAMI SP 10/92)^85^ with a maximum mean error of ±5 mmHg (±0.7kPa) and a maximum standard deviation of 8mmHg (1.1kPa). The accuracy of the blood pressure cuff is a significant limitation to using single-point or *hybrid calibration* for BP estimation. Slight errors in a single cuff reading, caused by instrumentation error as well as user error (movement, wrong cuff size, etc) may translate into a consistent offset in BP estimation. Consider, for example, 006 in Supplementary Information figure SI 5, the initial calibration during the rest period sets, with both $\Delta$SBP cuff and $\Delta$SBP est at 0 mmHg. In the following 5 cuff inflations, the $\Delta$SBP cuff readings decreased to just under -5mmHg, within the resolution of the ANSI/AAMI protocol. It is unclear whether this change in SBP is a real change (potentially caused by the participant relaxing after the start of the study) or if it was a result of instrumental errors in the blood pressure cuff. Either way, a consistent DC offset of 5-10mmHg was observed for the remaining BP estimates in this individual.

## Conclusion

Under an infusion of phenyleprhine, changes in the PPG (to a greater extent) and the ECG (to a lesser extent) reflect changes in BP that can be tracked using certain morphological features. For monitoring of BP by a single device, we recommend focusing on the PPG as this appears to be far superior to BP monitoring than using the ECG. In this study, we observed clear changes in the PPG in response to the dose increase of phenylephrine and characterised these by smooth muscle activation and a clear increase in the amplitude of the reflected tidal wave. These changes were mirrored in certain features and it appears that their relationship to $\Delta$BP may be non-linear. BP estimation using the PPG may offer similar performance to PAT which has significant limitations as it requires two synchronous devices (ECG and PPG) for accurate measurements. In general, the calibration protocol for accurate BP estimation requires more attention, especially if the relationship is non-linear. *Hybrid calibration* strategies may not adequately reflect the unique and individualised relationship between changes in BP and changes in the PPG. Therefore, they should be used with caution and only as a potential indicator of relative changes as opposed to a clinical assessment of BP.

## Supplementary Information

Supplementary Information.

## Data Availability

The datasets generated or analysed during the current study are not publicly available due to the sensitive and identifiable nature of our data, patient consent and restrictions of the ethics protocol to protect the privacy of patients involved in the study. Contact eoin.finnegan@eng.ox.ac.uk for any queries.
