# Supplementary material for: Features from the photoplethysmogram and the electrocardiogram for estimating changes in blood pressure
Source: Sci Rep. 2023 Jan 18;13:986. doi: 10.1038/s41598-022-27170-2 (PMC9849280; doi:10.1038/s41598-022-27170-2)
Supplement: Supplementary file 1 — Supplementary Information. [file 41598_2022_27170_MOESM1_ESM.pdf]

Features from the photoplethysmogram and the electrocardiogram for estimating changes in blood pressure - Supplementary Information

Eoin Finnegan[1,*], Shaun Davidson[1], Mirae Harford[1,2,3], Peter Watkinson[2,3], Lionel Tarassenko[1], and Mauricio Villarroel[1]

[1]Institute of Biomedical Engineering, Department of Engineering Science, University of Oxford, UK
[2]Critical Care Research Group, Nuffield Department of Clinical Neurosciences, University of Oxford
[3]NIHR Oxford Biomedical Research Centre, Oxford, UK.
[*]eoin.finnegan@eng.ox.ac.uk

SI: 1 Additional details of PPG principal components features

Figure SI 1 shows a visualisation of computed PCA eigenvectors (e.g. the visualisation of PPG PCA 1 is shown as the eigenvector corresponding to the largest eigenvalue in the covariance of Ψ_{PPG}).

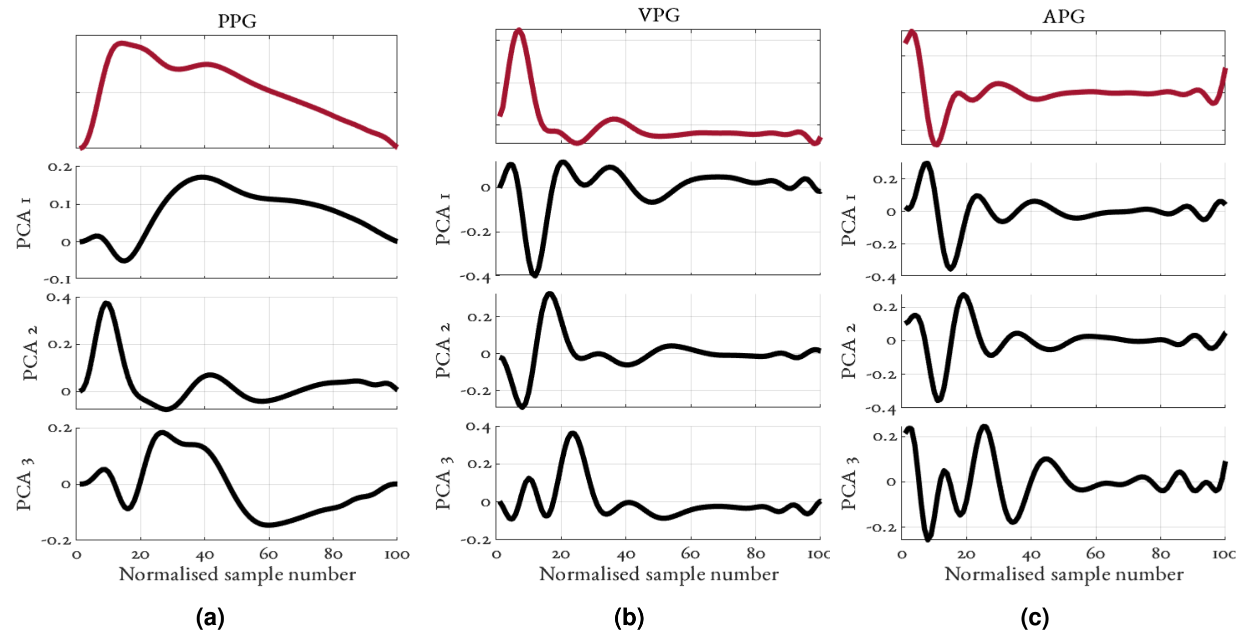

Figure SI 1. Visualisation of the 3 principal components of the (a) PPG, (b) VPG and (c) APG waveforms using the corresponding eigenvectors. The top row, in red, shows a typical PPG, VPG and APG beat (a-c respectively) in the dataset.

SI: 2 Additional details of ECG feature extraction

Complexity and entropy signals were extracted from each segmented ECG signal within the window w. For a given ECG segment, x, of length N_x, the following features were extracted:

Hjorth parameters The Hjorth mobility and complexity parameters indicate activity variations in a signal[1]. The mobility parameter represents the signal's mean frequency. The signal complexity is an estimate of the signal bandwidth. Hjorth mobility and complexity are computed as:

$$\text{Mobility} = \sqrt{\frac{\text{var}(x')}{\text{var}(x)}} \tag{SI 1}$$

$$\text{Complexity} = \frac{\text{Mobility}(x')}{\text{Mobility}(x)}. \tag{SI 2}$$

where var(·) denotes the variance.

Fractal dimension The fractal dimension of an ECG signal segment provides a measure of self-similarity. The fractal dimension of a time series is a number between 1 (straight line) and 2 (defining a surface), where a higher number represents a signal with higher fluctuations and complexity. To calculate the fractal dimension, we used the Higuchi algorithm[2] where increasing length scales (up to k_{max}) are used to estimate the signal length. The fractal dimension is then defined as the proportionality constant representing the relationship between length scale and signal length. Brezinski[3] showed that increasing values of k_{max} beyond a value of 17 resulted in only a marginal increase on the fractal dimension for regular ECG beats (defined as an ECG without arrhythmia episodes). Therefore, we set k_{max} as 17 in this work.

Shannon's entropy (SE) was used to measure the uncertainty of the information content in a time series based on it's probability distribution. The probability distribution of the time series was estimated by a normalised histogram. SE was then computed as:

$$\text{SE} = -\sum_{k=1}^{N_x} p_k \times \log(\frac{p_k}{w_k}) \tag{SI 3}$$

where p_k and w_k are the probability and width of the kth bin of the histogram.

Approximate entropy (approxEnt) quantifies the regularity of a time series and the likelihood that similar patterns of observations will not be followed by additional similar observations. For example, a time series containing many repetitive patterns has a small approxEnt. approxEnt was computed using the following steps:

1. Divide the signal, x, into consecutive segments of length $m = 2$. Following the work of Li[4], $m = 2$.
2. For each segment, i, compute the Chebyshev distance to all other segments and calculate C_i^m as the number of segments with a Chebyshev distance less than r to the i_{th} segment. Following the work of Li[4], $r = 0.2$.
3. Define $\phi^m(r)$ as the average number of segments of length m that are suitably similar to each other within a tolerance of r:

$$\phi^m(r) = \frac{1}{N_x - m + 1} \sum_{i=1}^{N_x-m+1} \ln C_i^m(r) \tag{SI 4}$$

4. To compare $\phi^m(r)$ to the subsequent data point, increase the dimension to m+1 and compute $\phi^{m+1}(r)$
5. The approximate entropy is computed as:

$$\text{approxEnt} = \phi^m(r) - \phi^{m+1}(r) \tag{SI 5}$$

Sample entropy (sampEnt) is a modification of approxEnt where each segment cannot be compared to itself. In approxEnt, the comparison between each segment and the rest of the segments also includes comparison with itself, as a result signals are interpreted to be more regular than they actually are. These self matches are not included in sampEnt resulting in a more stable estimate of entropy, reducing bias. We computed sampEnt using the same hyperparamters (m and r) as approxEnt.

Multi-scale entropy (MSE) is an extension to sample entropy. MSE is the application of sampEnt to the signal at increasingly coarser scales. For each sth scale, the original signal samples are grouped into non-overlapping windows, of length s, and the windowed samples are averaged. sampEnt is then applied to this averaged signal. We computed MSE at scales 2, 4, 6 and 8.

SI: 3 Results for mean arterial pressure and diastolic blood pressure

Table SI 1. Performance statistics of ΔMAP estimation using the models proposed. Results are given as median (IQR) computed across all folds of the LOSOCV. Entries in bold indicate the best performance for that metric.

**Model name**	ρ_p		**RMSE** *		**MAE** *	
Baseline reference	-	(-)	9.62	(4.67)	6.94	(3.97)
$\text{LASSO+OLS}_{\text{PPG}}$	0.81	(0.39)	6.17	(3.93)	4.77	(2.69)
RF_{PPG}	0.85	(0.21)	5.03	(3.97)	**4.05**	(**3.72**)
$\text{LASSO+OLS}_{\text{ECG}}$	0.69	(0.46)	6.67	(3.59)	5.53	(1.78)
RF_{ECG}	0.67	(0.46)	6.88	(2.16)	5.49	(1.49)
$\text{LASSO+OLS}_{\text{PPG+ECG}}$	0.80	(0.29)	6.33	(3.63)	4.73	(2.86)
$\text{RF}_{\text{PPG+ECG}}$	**0.86**	(**0.23**)	**4.94**	(**3.69**)	4.10	(3.77)
$\text{LASSO+OLS}_{\text{PAT}}$	0.77	(0.29)	6.87	(4.00)	5.14	(2.33)
RF_{PAT}	0.67	(0.24)	7.11	(3.39)	5.60	(2.77)

* results given in units of mmHg

Table SI 2. Performance statistics of ΔDBP estimation using the models proposed. Results are given as median (IQR) computed across all folds of the LOSOCV. Entries in bold indicate the best performance for that metric.

**Model name**	ρ_p		**RMSE** *		**MAE** *	
Baseline reference	-	(-)	8.82	(4.94)	6.82	(4.01)
$\text{LASSO+OLS}_{\text{PPG}}$	0.77	(0.31)	6.64	(4.79)	5.30	(3.97)
RF_{PPG}	0.84	(0.23)	**5.21**	(**3.65**)	4.54	(3.17)
$\text{LASSO+OLS}_{\text{ECG}}$	0.68	(0.44)	6.36	(3.86)	5.40	(3.05)
RF_{ECG}	0.59	(0.60)	7.06	(2.66)	5.28	(2.26)
$\text{LASSO+OLS}_{\text{PPG+ECG}}$	0.77	(0.52)	6.95	(4.10)	5.83	(3.40)
$\text{RF}_{\text{PPG+ECG}}$	**0.85**	(**0.24**)	5.39	(3.68)	**4.44**	(**3.11**)
$\text{LASSO+OLS}_{\text{PAT}}$	0.70	(0.41)	6.43	(4.23)	5.21	(2.93)
RF_{PAT}	0.63	(0.34)	7.00	(2.95)	5.24	(2.55)

* results given in units of mmHg

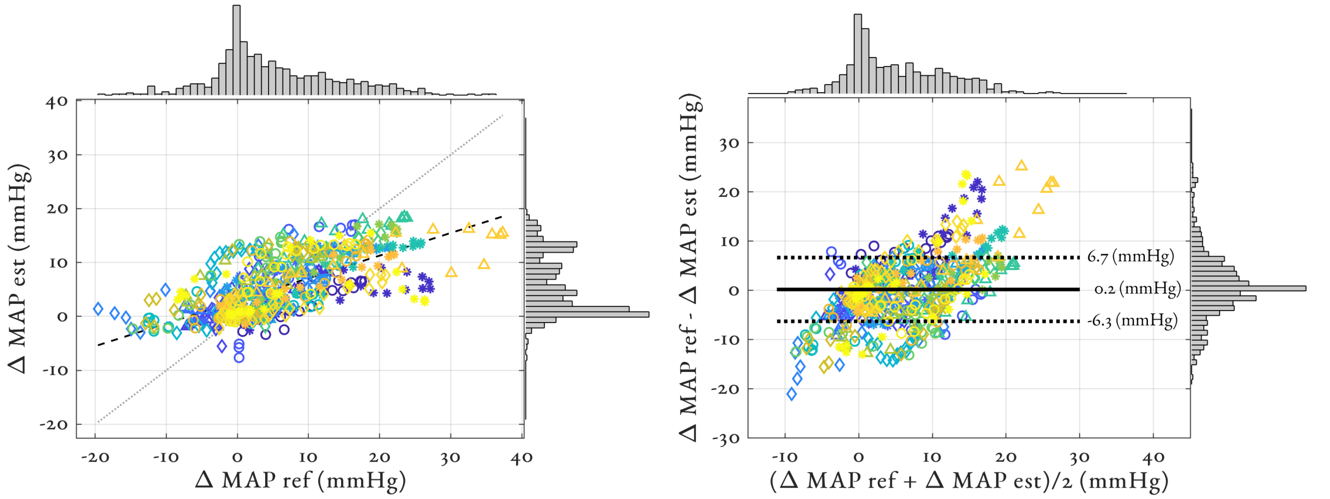

(a) (b)

Figure SI 2. Agreement between the reference ΔMAP values from the sphygmomanometer cuff and the estimated ΔMAP using $RF_{PPG+ECG}$. Individual participants are colour and marker-coded. (a) The correlation analysis, the overall correlation was 0.71, the median participant-wise correlation coefficient was 0.91 with a range of 0.31 to 0.97. Black striped line shows regression line. Grey dotted line shows line of unity. (b) The Bland-Altman analysis, the bias of the overall error was 0.2 mmHg with a standard deviation of 6.5 mmHg

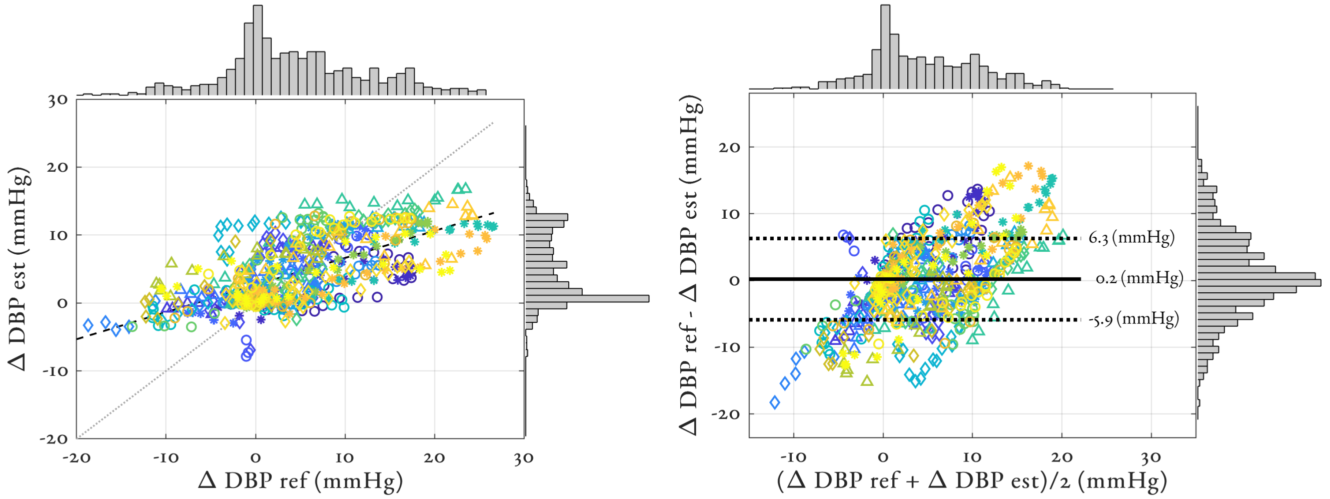

(a) (b)

Figure SI 3. Agreement between the reference ΔDBP values from the sphygmomanometer cuff and the estimated ΔDBP using $RF_{PPG+ECG}$. Individual participants are colour and marker-coded. (a) The correlation analysis, the overall correlation was 0.67, the median participant-wise correlation coefficient was 0.86 with a range of 0.42 to 0.89. Black striped line shows regression line. Grey dotted line shows line of unity. (b) The Bland-Altman analysis, the bias of the overall error was 0.2 mmHg with a standard deviation of 6.2 mmHg

SI: 4 Feature results

Table SI 3 provides a summary of the 45 features remaining after removing collinear features and the features from the original set with which they best correlate (defined as $|\rho_p| > 0.8$ $p < 0.05$). The overall correlations of these features to ΔSBP across the cohort was in general quite low with only one feature ($\text{Gauss}_{\sigma 4/A1}$) having $|\rho_{p\Delta\text{SBP}}| > 0.5$. There were a number of features that had significant participant-wise correlations (PWC) to ΔSBP, 21 features with a median absolute PWC > 0.5.

Table SI 3. Resulting features from removing collinearity and their correlated features ($|\rho_p| > 0.8$, $p < 0.05$). $\rho_{p\Delta\text{SBP}}$: correlation to Δ SBP across all participants, p: resulting p value. Features are ordered by their median SBP RF ranking coefficient. As demographics were included as static variables to the feature set, their correlations are not included.

**Feature name**	**Correlated features**	$\rho_{p\Delta\text{SBP}}$	p	**PWC [q1, q3]**
Kurtosis		0.42	< 0.001	[-0.11, 0.92]
σ_{g1}	$Gauss_{sys/dias}$; Gauss RTT; μ_{g1}; μ_{g2}; σ_{g2}; μ_{g3}	-0.25	< 0.001	[-0.9, -0.49]
$Gauss_{\sigma 4/A1}$	PPG AI; $Gauss_{A4/A1}$; A_{g4}; μ_{g4}; σ_{g4}	0.51	< 0.001	[0.57, 0.92]
Hjorth mobility		-0.42	< 0.001	[-0.85, -0.47]
VPG PCA_1		-0.18	< 0.001	[-0.88, 0.04]
APG PCA_1		-0.11	< 0.01	[-0.91, -0.12]
e / a		0.21	< 0.001	[-0.03, 0.76]
A_{g2}		-0.25	< 0.001	[-0.74, -0.28]
c / a		0.02	0.576	[0.16, 0.84]
Gauss AI		0.15	< 0.001	[-0.81, -0.2]
$slope_{bd}$	AGI	-0.11	< 0.01	[-0.75, -0.06]
VPG PCA_3		-0.32	< 0.001	[-0.79, 0.2]
IPA	T_{Dia}; N_{amp}; RI; A2; sVRI; Sys_{μ}	0.3	< 0.001	[0.26, 0.85]
Gauss LVET		0.32	< 0.001	[-0.19, 0.55]
A_{g3}		-0.18	< 0.001	[-0.63, -0.02]
IHAR	T_{Sys}; T_{Dia}; T_{Ratio}; A1	-0.3	< 0.001	[-0.8, -0.38]
Gauss RI_R	A_{g1}	0.41	< 0.001	[0.29, 0.72]
Fractal dimension		-0.41	< 0.001	[-0.83, -0.13]
σ_{g3}	μ_{g4}	-0.31	< 0.001	[-0.79, -0.29]
Dia_{σ}		0.13	< 0.01	[-0.65, 0.3]
BP calibration		–	–	[–, –]
Hjorth complexity		0.21	< 0.001	[-0.07, 0.48]
MSE scale 8	MSE scale 4; MSE scale 6	-0.28	< 0.001	[-0.77, -0.2]
$Width_{50}$	$Width_{25}$	0.17	< 0.001	[-0.32, 0.75]
Height		–	–	[–, –]
BMI	Weight	–	–	[–, –]
PPG PCA_3		0.06	0.121	[-0.68, -0.07]
Gauss RI		0.23	< 0.001	[-0.12, 0.71]
sampEnt	MSE scale 2; MSE scale 4	-0.01	0.789	[-0.55, 0.1]
d / a		-0.18	< 0.001	[0.5, 0.88]
APG PCA_2		0	0.933	[-0.67, 0.43]
NHA	N_{amp}; Sys_{μ}	0.12	< 0.01	[0.11, 0.73]
approxEnt		-0.17	< 0.001	[-0.78, -0.21]
Age		–	–	[–, –]
Skewness		-0.05	0.212	[-0.57, 0.58]
b / a	A_{g1}	-0.25	< 0.001	[-0.72, 0.19]
VPG PCA_2		0.11	< 0.01	[-0.65, 0.3]
APG PCA_3		0.09	< 0.05	[-0.69, 0.56]
PPG PCA_2		-0.02	0.695	[-0.62, 0.25]
STT	CT	0.09	< 0.05	[-0.55, 0.36]
Gauss AI_R		-0.08	0.052	[-0.56, 0.47]
PPG PCA_1		-0.16	< 0.001	[-0.72, 0.01]
Sys_{σ}		-0.03	0.469	[-0.29, 0.52]
SE		0.02	0.527	[-0.42, 0.33]
Sex		–	–	[–, –]

PWC: participant-wise correlation, q1 and q3 are the first and third quartile PWV values across the cohort.

Figure SI 4 shows median SBP, MAP, and DBP ranking coefficients for both (a) RF and (b) LASSO+OLS SHAP values feature importance. We quantified the agreement between the feature ranks for pairs of SBP, MAP, and SBP using the Kendall rank correlation coefficient, ρ_k[5]. It was found that the feature importance for SBP, MAP, and DBP estimation showed strong agreement with each other ($\rho_k > 0.6$ for all).

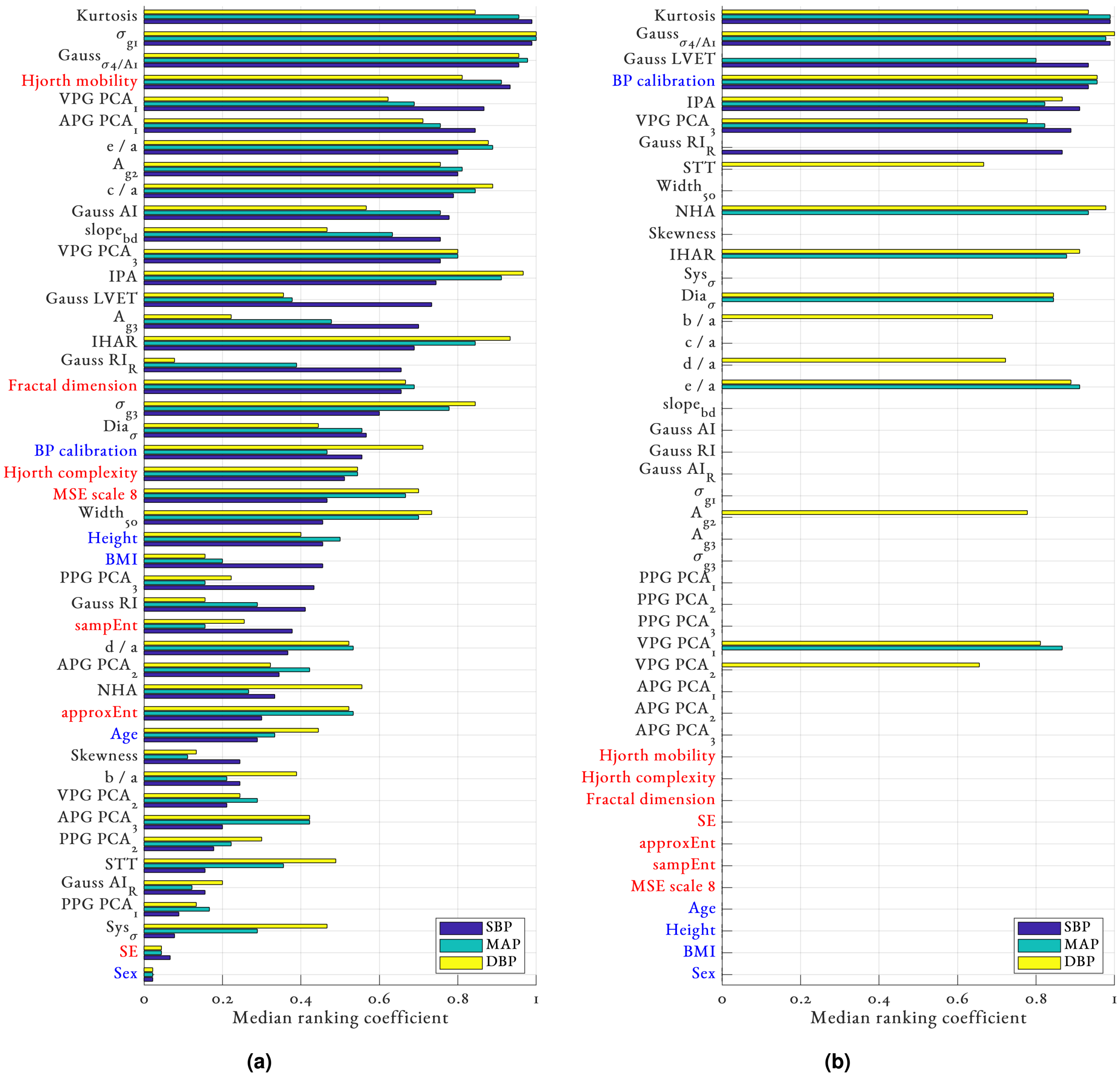

Figure SI 4. Median SBP, MAP, and DBP SHAP values feature ranking coefficient for (a) random forest and (b) LASSO+OLS both with PPG + ECG feature set. Features are ordered by their respective median ranking coefficient for SBP. Demographic features are highlighted in blue and ECG features in red for clarity.

SI: 5 Individual results

Figures SI 5 to SI 7 shows the individual results for blood pressure estimation using the $RF_{PPG+ECG}$ model across all participants in the study. Under each participant we show the summed SHAP values for each observation grouped in to PPG features, ECG features and demographics. SHAP values in red indicate observations where a significant accumulated marginal contribution was reported. Features from the PPG consistently offered the largest contribution to blood pressure estimation.

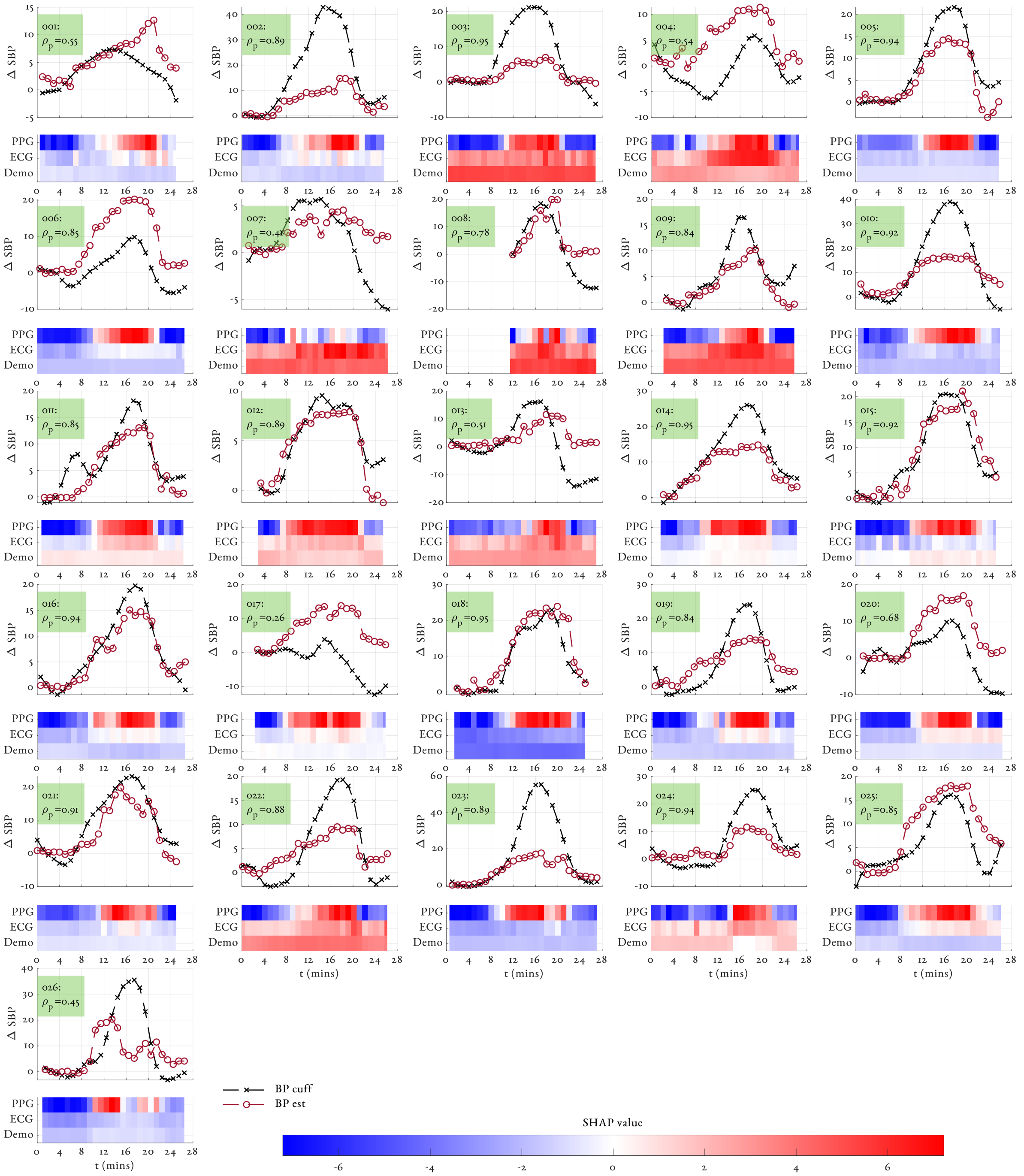

Figure SI 5. Individual results of ΔSBP estimation using the $RF_{PPG+ECG}$ model across all participants in the study. Note the differences in the y-axis. The reference Δ SBP values from the cuff are shown in black and the estimated values are shown in red. Under each participant we show the summed SHAP values for each observation grouped in to PPG features, ECG features and demographics.

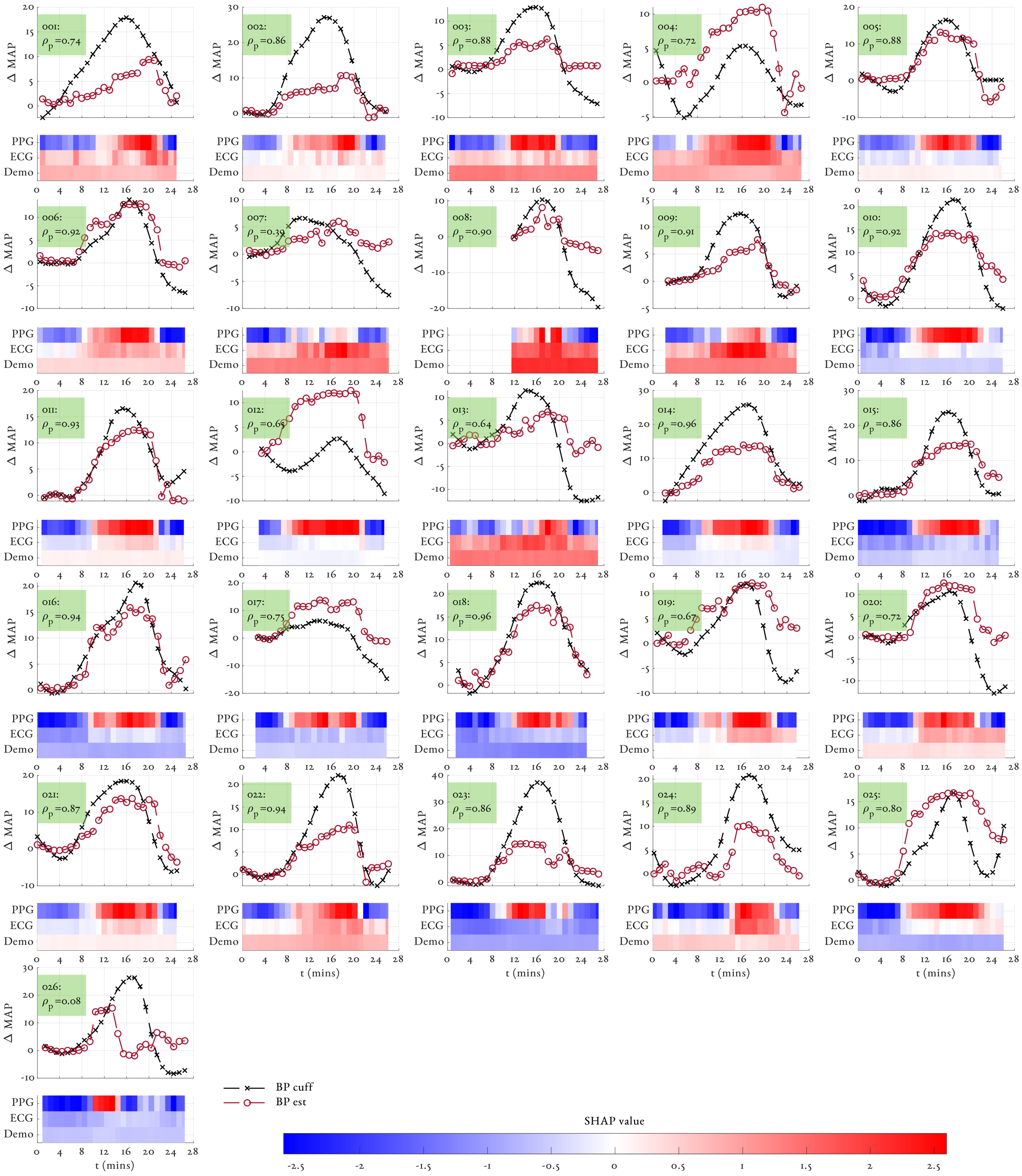

Figure SI 6. Individual results of ΔMAP estimation using the $RF_{PPG+ECG}$ model across all participants in the study. Note the differences in the y-axis. The reference Δ MAP values from the cuff are shown in black and the estimated values are shown in red. Under each participant we show the summed SHAP values for each observation grouped in to PPG features, ECG features and demographics.

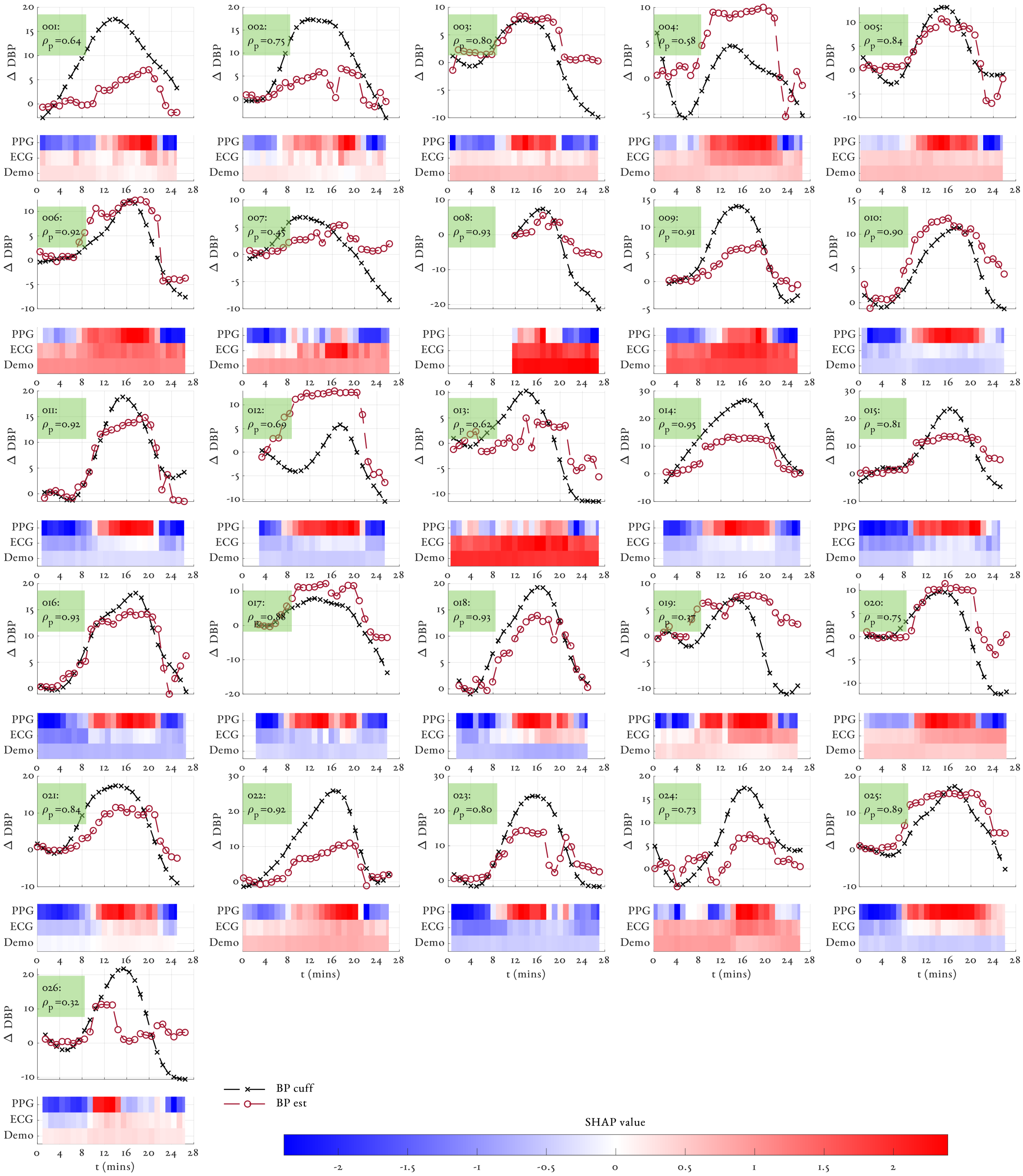

Figure SI 7. Individual results of ΔDBP estimation using the $RF_{PPG+ECG}$ model across all participants in the study. Note the differences in the y-axis. The reference Δ DBP values from the cuff are shown in black and the estimated values are shown in red. Under each participant we show the summed SHAP values for each observation grouped in to PPG features, ECG features and demographics.

SI: 6 Individual changes in cardiac output

Figure SI 8 shows the changes in observed for all individuals in the dataset across the full duration of their session. The majority of individuals experienced a decrease in driven by a decrease in HR.

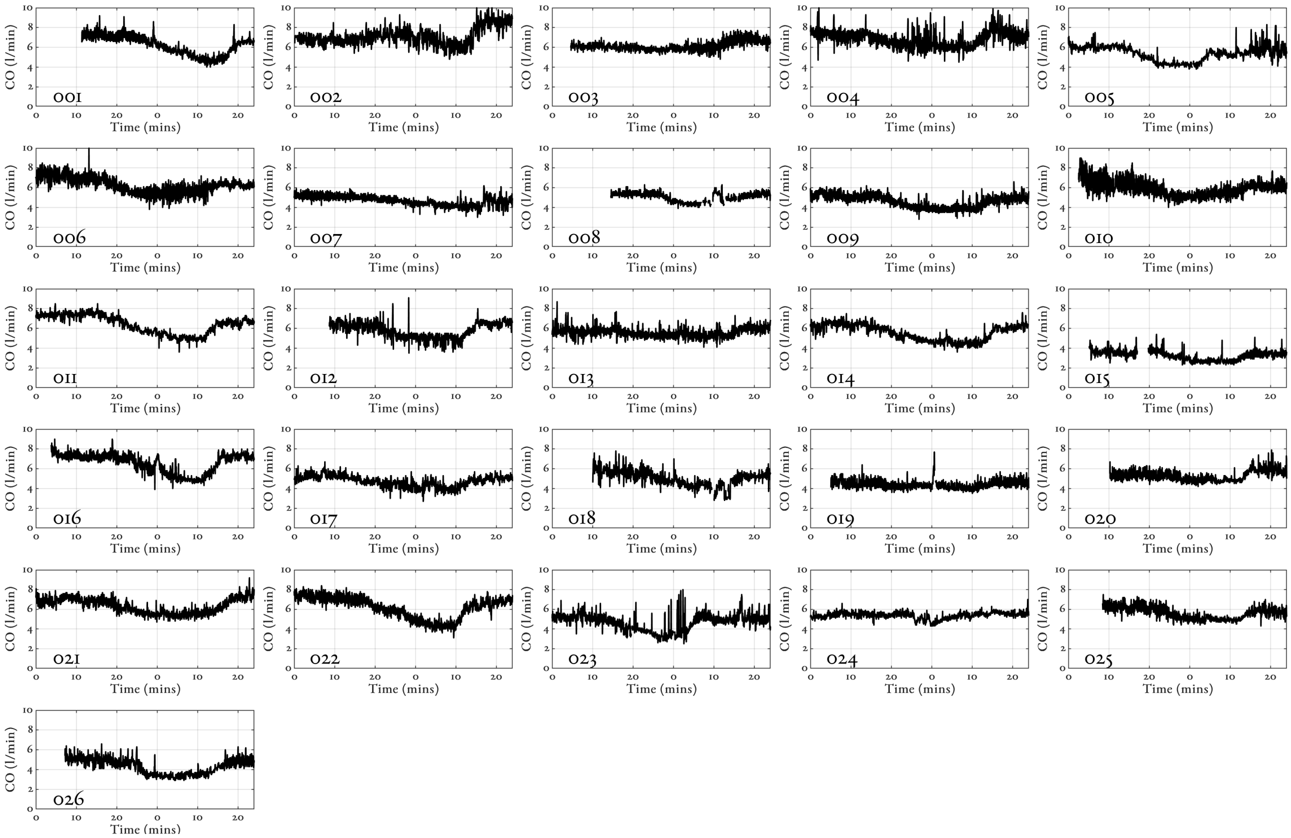

Figure SI 8. Individual changes in CO observed in all individuals across the full duration of their session.

References

1. Rizal, A. & Hadiyoso, S. ECG signal classification using Hjorth Descriptor. In *2015 International conference on automation, cognitive science, optics, micro electro-mechanical system, and information technology (ICACOMIT)*, 87–90 (IEEE, 2015).
2. Higuchi, T. Approach to an irregular time series on the basis of the fractal theory. *Phys. D: Nonlinear Phenom.* **31**, 277–283 (1988).
3. Brezinski, K. ECG Beat Classification Using Higuchi Fractal Dimension and Heart Rate Variability. Tech. Rep., University of Manitoba (2019).
4. Li, H. *et al.* A new ECG signal classification based on WPD and ApEn feature extraction. *Circuits, Syst. Signal Process.* **35**, 339–352 (2016).
5. Kendall, M. G. A new measure of rank correlation. *Biometrika* **30**, 81–93 (1938).
